# Sense and antisense transcription are associated with distinct chromatin architectures across genes

**DOI:** 10.1093/nar/gkv666

**Published:** 2015-06-29

**Authors:** Struan C. Murray, Simon Haenni, Françoise S. Howe, Harry Fischl, Karolina Chocian, Anitha Nair, Jane Mellor

**Affiliations:** Department of Biochemistry, University of Oxford, South Parks Road, Oxford, OX1 3QU, UK

## Abstract

Genes from yeast to mammals are frequently subject to non-coding transcription of their antisense strand; however the genome-wide role for antisense transcription remains elusive. As transcription influences chromatin structure, we took a genome-wide approach to assess which chromatin features are associated with nascent antisense transcription, and contrast these with features associated with nascent sense transcription. We describe a distinct chromatin architecture at the promoter and gene body specifically associated with antisense transcription, marked by reduced H2B ubiquitination, H3K36 and H3K79 trimethylation and increased levels of H3 acetylation, chromatin remodelling enzymes, histone chaperones and histone turnover. The difference in sense transcription between genes with high or low levels of antisense transcription is slight; thus the antisense transcription-associated chromatin state is not simply analogous to a repressed state. Using mutants in which the level of antisense transcription is reduced at *GAL1*, or altered genome-wide, we show that non-coding transcription is associated with high H3 acetylation and H3 levels across the gene, while reducing H3K36me3. Set1 is required for these antisense transcription-associated chromatin changes in the gene body. We propose that nascent antisense and sense transcription have fundamentally distinct relationships with chromatin, and that *both* should be considered canonical features of eukaryotic genes.

## INTRODUCTION

Non-coding transcription on both strands of DNA is a genomic feature of all three kingdoms (1). While many non-coding transcripts have regulatory functions (2), other non-coding transcripts, particularly those transcribed from the antisense strand of protein-coding genes, are often rapidly degraded (3,4), leading to underestimates in the extent of antisense transcription genome-wide (5), and the proposal that they may serve no meaningful biological function (6). Genome-wide measurements of *nascent* transcription in yeast (7,8), as opposed to transcript levels, give a very different view of antisense transcription, both in terms of its abundance and its relationship with sense (pre-mRNA) transcription (9). Firstly, there is *no* correlation between levels of nascent sense and antisense transcription at individual genes (*r*_s_ = −0.06) (9). Secondly, antisense transcription is a relatively frequent event. The average ratio of nascent sense to antisense transcription across a gene is 5:1 for genes without a defined antisense transcript, and 5:2 for those with a defined transcript (9). That antisense transcription is so abundant leads one to wonder whether this might have any effect upon chromatin structure, particularly at promoters or over transcribed regions.

Promoters are marked by a defined, nucleosome depleted region (NDR), in which the transcription machinery and transcription factors bind, flanked by the −1 and +1 nucleosomes (10). These promoter-associated nucleosomes often contain the variant histone H2A.Z (11), exhibit a high rate of incorporation of newly-synthesized histone H3 (12,13), and are enriched for H3 acetylation (14) and Set1-dependent trimethylation of lysine 4 (H3K4me3) (15), independently of histone turnover (13). The gene body (the region between the sense promoter and terminator) also contains unique chromatin features, which could conceivably be influenced by antisense transcription. Set2-dependent H3K36 trimethylation is found within the gene body and is positively correlated with gene activity (14). At *STE11*, H3K36me3 may suppress co-transcriptional turnover of nucleosomes (16). Genome-wide, H3K36me3 is necessary for the recruitment of the Rpd3S deacetylase complex (containing Eaf3 and Rco1) through which it is thought to maintain the gene body in a hypoacetylated state (17,18), and chromatin remodelling enzymes such as Isw1 and Chd1 (19). However, H3K36me3 and histone turnover are now known to be two correlating but largely independent events in the gene body (13). H3K79me3 and H2BK123 ubiquitination are also found within the gene body, and appear to mark stable nucleosomes (20,21).

Given that a gene subject to both sense and antisense transcription has two overlapping convergent transcription units, and since antisense transcription generally extends into the 5′ sense promoter (7,8), it is commonly assumed that antisense transcription will lead to H3K36 methylation and/or histone deacetylation around the sense promoter, stabilizing or remodelling nucleosomes and repressing transcription initiation. Indeed, some regulatory long non-coding RNAs are reported to use this strategy to repress gene expression (22–24). However, at the genome-wide level this is at odds with the lack of correlation between sense and antisense transcription (9).

Here we ask how antisense transcription influences chromatin around the sense promoter and across the gene body. Using extensive correlations of genome-wide data sets, we find that nascent sense and antisense transcription are each associated with distinct features of chromatin. Antisense transcription is associated with increased levels of chromatin remodelling enzymes and histone chaperones at the sense promoter, increased nucleosome occupancy and closure of the NDR. Across the gene body, antisense transcription is associated with an increase in histone acetylation and a reduction in levels of H3K36me3, H3K79me3 and H2BK123 ubiquitination. Remarkably, antisense transcription is also associated with increased histone turnover at the sense promoter and gene body. All these changes occur in a stepwise manner across five gene groups with different levels of antisense transcription, thus even small changes in antisense transcription level are associated with changes in chromatin. Using an experimental system in which antisense transcription across the *GAL1* gene is reduced, we demonstrate that loss of antisense transcription causes expected changes to the chromatin. To validate these links between chromatin modifications and antisense transcription, we used mutant yeast strains with altered levels of antisense transcription around the sense promoter and show that an increase in antisense transcription is concurrent with increased histone acetylation, increased nucleosome occupancy and decreased H3K36me3. We propose that sense and antisense transcription define distinct chromatin architectures, and that these will have distinct effects on gene behaviour.

## MATERIALS AND METHODS

### Classification of genes

Transcription start (TSS) and end sites were annotated as described previously (9). If no site was available, a hypothetical TSS was assigned using a genome-wide consensus. Genes defined as dubious or which overlapped considerably were removed from all analyses. Remaining genes (5183) were divided into five classes, each comprising approximately 20% of the total, based on levels (sequence reads) of strand-specific nascent transcription, determined previously by NET-seq (7), in a 300 bp window downstream of the sense TSS. Genes were defined as TATA-box containing or TATA-less using the classifications by (25).

### Genome-wide ChIP data

Genome-wide levels of all proteins were obtained from sources described in the supplementary experimental procedures. *All* H3 modifications were normalised to H3 levels before subsequent analysis. To assess the correlation of a given feature with sense and antisense transcription, the level of the feature inside a 10 bp window slid from −1000 bp to +1500 bp relative to the TSS was determined for 5183 genes described in the main text, and the Spearman's correlation coefficient determined for each window step.

### Transcription machinery at promoters

The occupancies of 202 different transcription-related proteins at gene promoters were determined using a comprehensive ChIP-chip study in TAP-tagged yeast strains (10). *P*-values were determined using the Wilcoxon rank-sum test and comparing the distribution of occupancy levels between two gene groups. Further details can be found in the supplementary information.

### Nucleosome occupancy and NDR sizes

Nucleosome occupancy levels and coordinates were determined as described previously (26). The distance between the −1 and +1 nucleosomes was defined as the NDR (nucleosome depleted region) size for a given gene determined using these coordinates, and the median size for a given group calculated.

### Histone turnover data

Estimated levels of H3 turnover genome-wide were obtained from (12) and (16). At a given gene region (−1 to +4 nucleosomes), the H3 turnover rate was determined as the maximum rate of all the probes overlapping that region. To correlate histone turnover with histone modification genome-wide, the average level of a given modification was determined within every probe for which turnover estimates were available. A Spearman's correlation coefficient was then determined by comparing levels of modification with histone turnover.

### Genome-wide correlations with nascent transcription

The levels of numerous modifications were determined within a sliding 50 bp window moved across the transcribed regions for 5183 genes. The modification levels within these windows were then correlated with the level of nascent sense and, separately, antisense transcription in the same windows, in order to calculate a Spearman's correlation coefficient.

### Grouping genes according to changes in antisense transcription following *SET2, RCO1, EAF3* or *SET1* deletion

**Figure 1.**
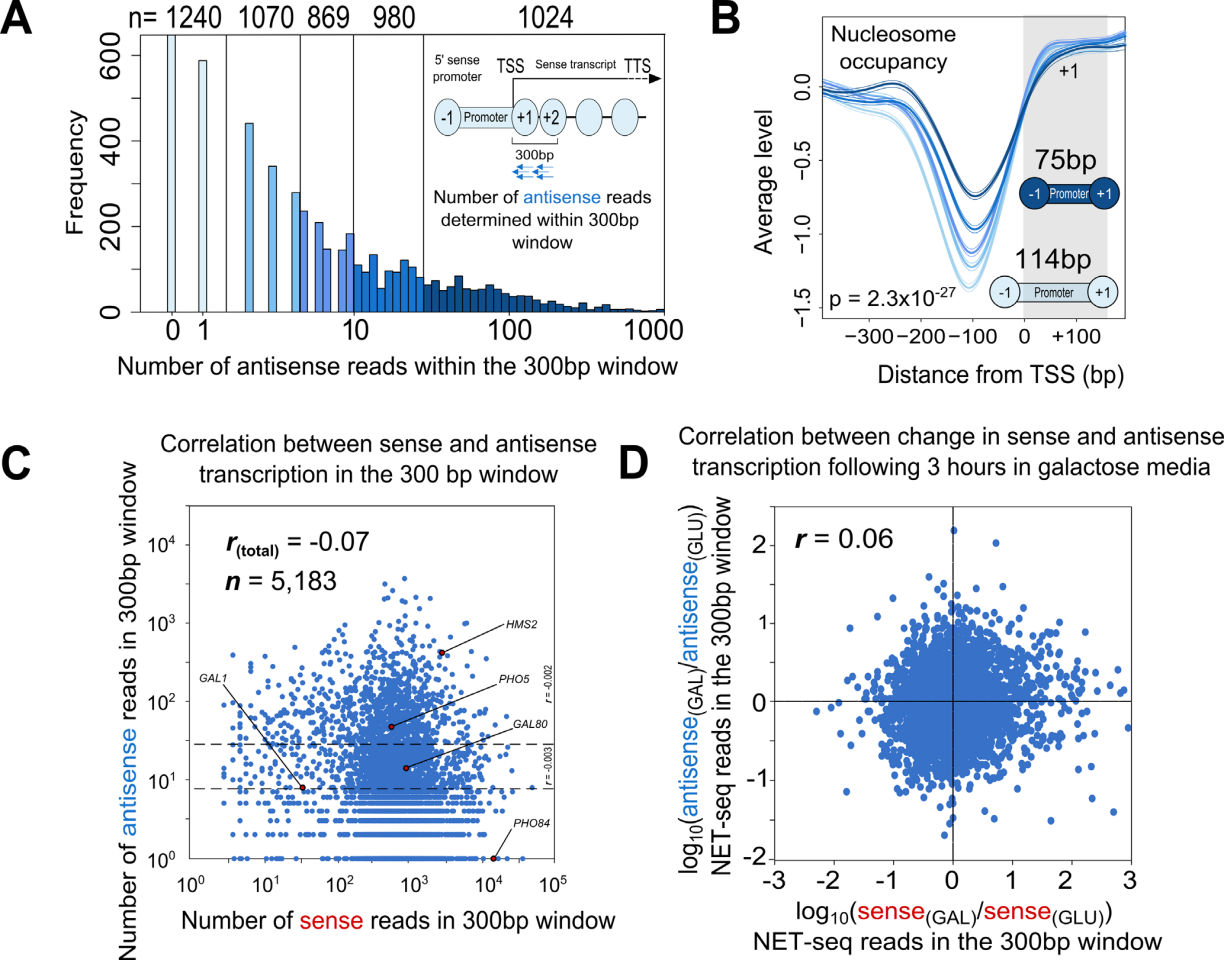
Antisense transcription is associated with an increase in nucleosome occupancy at sense promoters, but not with sense transcription. (**A**) The genome-wide distribution of antisense transcription NET-seq reads in a 300 bp window immediately downstream of the transcription start site (see inset), divided into five colour-coded classes. Shown are the 5183 yeast genes used in this study. (**B**) Average levels of nucleosome occupancy around the TSS (0) in the five classes shown in A, together with the NDR sizes of the top and bottom classes (see inset). The grey rectangle represents the approximate position of the +1 nucleosome. (**C**) A scatter plot of the number of antisense NET-seq reads in the window described in A, versus the number of sense NET-seq reads within the same window. (**D**) Correlation between sense and antisense transcription across genes in cells transferred from glucose- to galactose-containing medium for 3 hours. .

For each mutant, three gene groups were drawn from the 5183 genes defined above, dependent on whether a given gene's antisense transcription in the 300 bp window increased, decreased or was unchanged in the mutant relative to wild-type. The NET-seq data used were those from (7), and were normalised as described there such that the total read count in wild-type, *set2Δ*, *rco1Δ, eaf3Δ* and *set1Δ* strains was the same. Every gene was then assigned to one of the five antisense transcription classes presented in Figure 1A, for each of the five strains. For a given deletion strain, a gene was defined as ‘increased’ if it went up by at least two classes in the mutant relative to the wild-type, ‘decreased’ if it went down by at least two, and ‘unchanged’ if it did not change classification. Details on the mapping and normalisation of ChIP-seq data in the same deletion strains can be found in the supplementary information.

### Statistical tests

All *P*-values were calculated using the Wilcoxon rank sum test. For Figs displaying the average levels of a given factor or modification around the TSS, *P*-values shown are those calculated by comparing the distributions at the maximum point in the vicinity of the TSS for the highest and the lowest antisense-transcribed groups, or at the minimum points for graphs of nucleosome occupancy, H3K36me3, H3K79me2, H3K79me3 and H2BK123ub.

### Strain construction

*GAL1* strains used were modified versions of those described previously (9), with additional modifications described in Supplementary Methods.

### Northern blotting

Northern blotting was carried out as described previously (9).

### Chromatin immunoprecipitation

ChIP was carried out as described previously (27).

## RESULTS

### Promoters subject to antisense transcription show increased levels of chromatin remodelling enzymes and histone chaperones.

To address how antisense transcription impacts on the canonical features around the sense promoter, we defined all genes based on the level of *nascent* antisense transcription in a 300 bp window immediately downstream of the sense promoter, excluding any overlapping, convergent genes (Figure 1A). This allowed us to examine how sense promoters subject to high levels of antisense transcription differ from those with lower levels.

We divided 5183 *S. cerevisiae* genes into five classes based on the number of NET-seq reads in the 300 bp window. This gave us a class of 1024 genes (20%) with high levels of antisense transcription (≥28 reads, median 70 reads), a class of 1240 genes (24%) with little or no antisense transcription (0 or 1 read), and three intermediate classes (Figure 1A). We then assessed whether genes in the class with the highest antisense transcription were enriched for specific factors when compared to those in the lowest, utilizing an extensive analysis in which the levels of 202 promoter factors were determined genome-wide (10). Strikingly, we found that the promoters of genes subject to high levels of antisense transcription were significantly enriched for factors involved in modulating the chromatin environment (Table 1; Supplementary Table S1). To assess whether these enriched factors were a unique feature of genes subject to antisense transcription, or just transcribed genes in general, we compared the group with highest antisense transcription (median 70 antisense reads, 578 sense reads, *n* = 1024) with another group which had no or low levels of antisense transcription (0 or 1 read) but higher levels of sense transcription (median 948 reads, *n* = 1240) within the same 300 bp window (Supplementary Table S2). Components of the ISW1, ISW2 and INO80 remodelling complexes and the FACT histone chaperone (28) were specifically enriched at the sense promoters of genes subject to high antisense transcription compared to gene promoters with high levels of sense transcription (Table 1). Thus, antisense transcription is likely to be associated with specific chromatin remodelling enzymes and changes in promoter chromatin structure.

**Table 1.** Antisense transcribed genes are enriched for distinct promoter-bound transcription related proteins

Enriched factor	Complex	Function	*P*-value^a^
Isw1	Isw1a, Isw1b	Chromatin remodelling enzyme	4.2 × 10^−9^
Ino80	INO80	Chromatin remodelling enzyme	4.9 × 10^−9^
Pob3	FACT	Histone chaperone	2.5 × 10^−7^
Rsc9	RSC1, RSC2, RSCa	Chromatin remodelling enzyme	3.2 × 10^−7^
Swi3	SWI-SNF	Chromatin remodelling enzyme	4.9 × 10^−7^
Rpd3	RPD3	Histone chaperone and lysine deacetylase	2.9 × 10^−6^
Rpb7	Pol II	Recruitment of 3′-end processing factors	3.8 × 10^−6^
Itc1	Isw2	Component of chromatin remodelling complex	1.2 × 10^−5^
Spt3	SAGA,SLIK	Negative acting subunit of the SAGA and SAGA-like transcriptional regulatory complexes	1.9 × 10^−5^
Ctk1	CTK	CTD phosphorylation, regulates mRNA 3′ end processing	2.1 × 10^−5^
Spn1 (Iws1)	SPT6 interactor	Interacts with RNAP II, TBP and chromatin remodelling factors	2.2 × 10^−5^
Spt6	SPT6	Histone chaperone	2.9 × 10^−5^
Rpo21	Pol II	Largest Pol II subunit	3.8 ×10^−5^
Htb2	nucleosome	Core histone	6.7 × 10^−5^
Rpb2	Pol II	Second largest Pol II subunit	8.8 × 10^−5^

^a^Factors are ranked in order of *P*-values determined using the Wilcoxon rank sum test, and were considered enriched if their levels were higher in those genes with high antisense compared to low antisense and if they had a *P*-value less than 0.0001. Factors in blue were significantly enriched in genes with antisense transcription compared to those with sense transcription but no antisense transcription (see Supplementary Table S2).

### Antisense transcription is associated with a change in chromatin architecture at the sense promoter

We asked how genes might differ in the positioning, dynamics, and occupancy of their promoter-bound nucleosomes. Using a genome-wide map of nucleosome occupancy (29), we found that genes subject to high levels of antisense transcription showed elevated nucleosome occupancy across their promoters (*p* = 2.3 × 10^−27^; Figure 1B). The NDR between the −1 and +1 nucleosomes was also shorter in genes with high antisense transcription (Figure 1B; a median length of 75 bp versus 114 bp for the highest and lowest classes respectively, *p* = 1 × 10^−16^). The increase in nucleosome occupancy occurred in a stepwise fashion between the five gene classes, suggesting that antisense transcription can exert changes in the chromatin even at low levels and in ≈75% of genes. Critically, we found that there is only a very weak correlation between sense and antisense transcription within the 300 bp window (Figure 1C, Spearman's correlation coefficient = −0.07). Furthermore, changes in sense transcription were not found to be inversely correlated with changes in antisense transcription genome-wide using NET-seq experiments conducted in cells shifted from glucose- to galactose-containing medium, despite there being a >3-fold change in sense transcription for 1078 genes (20% of the genome) (8) (Figure 1D, *r*_s_ = +0.06). Thus the increase in nucleosome occupancy associated with increasing antisense transcription is likely to be *independent* of sense transcription.

### Sense and antisense transcription are associated with distinct patterns of nucleosome occupancy at the sense promoter

**Figure 2.**
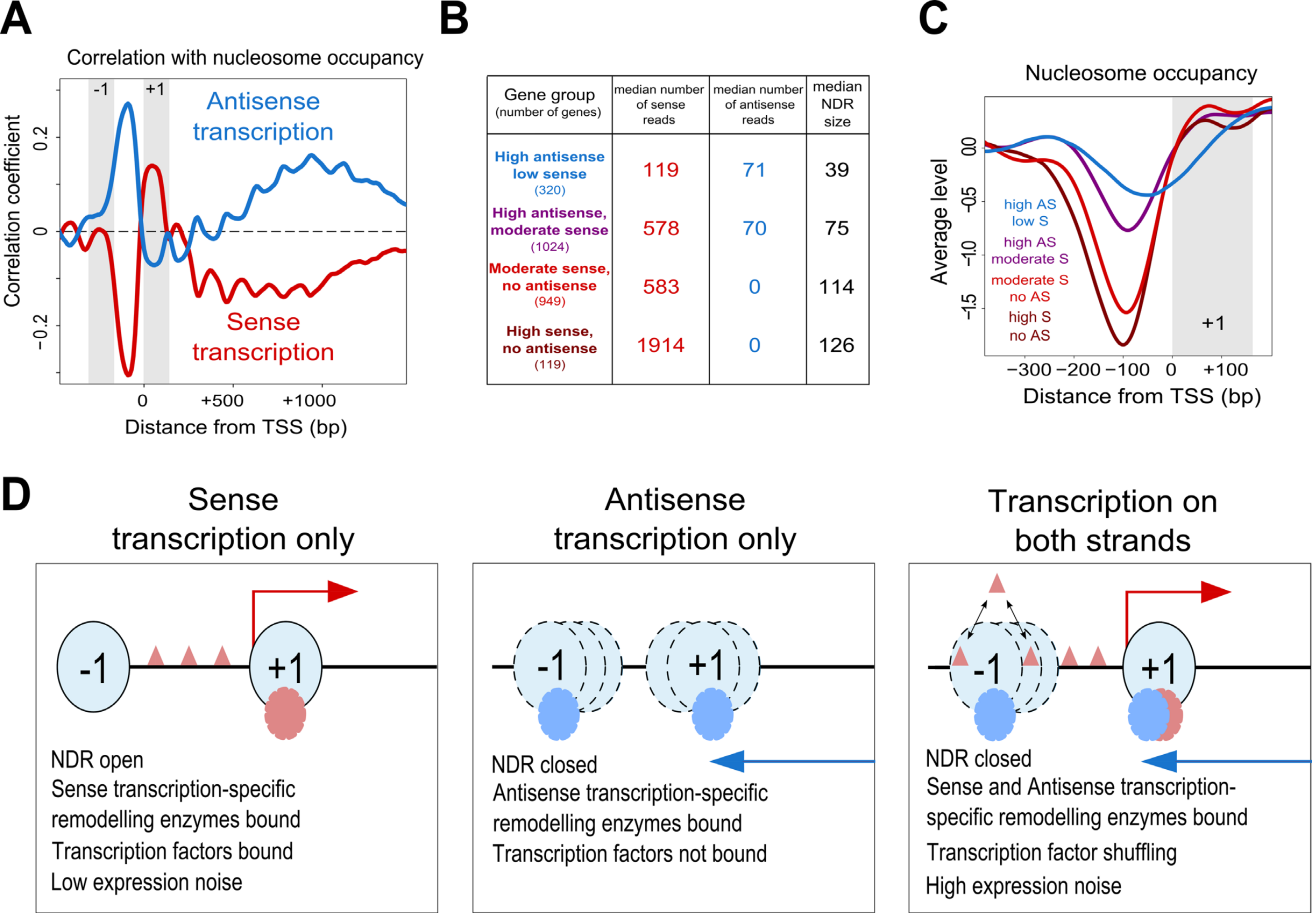
(**A**) Correlation between nucleosome occupancy and the two types of transcription. Shown is the Spearman correlation coefficient calculated for sense transcription (red) and, separately, antisense transcription (blue) in the 300 bp window with nucleosome occupancy at varying positions around the TSS. Grey rectangles represent the approximate positions of the −1 (left) and +1 (right) nucleosomes. (**B**) Median number of sense and antisense reads in the gene groups shown in panel (C), together with their median NDR size. (**C**) Average nucleosome occupancy in genes with varying median numbers of sense and antisense reads in the same window, with colours referring to the gene groups described in panel (B). (**D**) Promoters can be considered to belong to one of three different classes under a given set of conditions, each with distinct chromatin features. Triangles represent transcription factors, ovals chromatin remodelling enzymes. See Supplementary Figures S1 and S2.

Next, we assessed how nascent sense transcription in the same 300 bp window influences nucleosome occupancy and NDR size at the sense promoter in the presence of varying levels of antisense transcription (Figure 2A–D). First, we asked how the levels of sense or antisense transcription in the 300 bp window correlated with nucleosome occupancy at varying positions relative to the TSS (Figure 2A). We found that sense transcription was negatively correlated with nucleosome occupancy immediately upstream of the TSS, and positively correlated immediately downstream, in the region corresponding to the +1 nucleosome, supporting a model in which sense transcription positions the +1 nucleosome (30). In contrast, antisense transcription correlated positively with nucleosome occupancy over the promoter, directly upstream of the TSS, and in the first 1 kb of the transcribed region. Genes with sense transcription but no antisense transcription tended towards an open promoter chromatin structure, with low nucleosome occupancy and a large NDR (126 bp), while genes with high antisense transcription and low sense transcription had a closed promoter chromatin structure with high nucleosome occupancy across the promoter and a small NDR (39 bp) (Figure 2B and C). We confirmed that the increased nucleosome occupancy associated with antisense transcription is independent of sense transcription by comparing genes with similar levels of sense transcription but varying levels of antisense transcription, and found that nucleosome occupancy still differed markedly (Figure 2B and C). This suggests three distinct promoter chromatin architectures, defined by genes enriched primarily for sense transcription (Figure 2D left panel), primarily for antisense transcription (middle panel), or genes with varying levels of both sense and antisense transcription (right panel). These architectures are highly reminiscent of the distinct classes of condition-specific promoter chromatin configurations associated with different degrees of gene expression noise (26). Thus, sense and antisense transcription are associated with distinct properties of the chromatin organization surrounding the sense TSS. Although sense and antisense transcription are inversely associated with nucleosome occupancy, the very weak correlation between sense and antisense transcription genome-wide suggests that they are largely independent processes, where one does not regulate the levels of the other.

### Nascent sense and antisense transcription are associated with distinct patterns of histone modification

**Figure 3.**
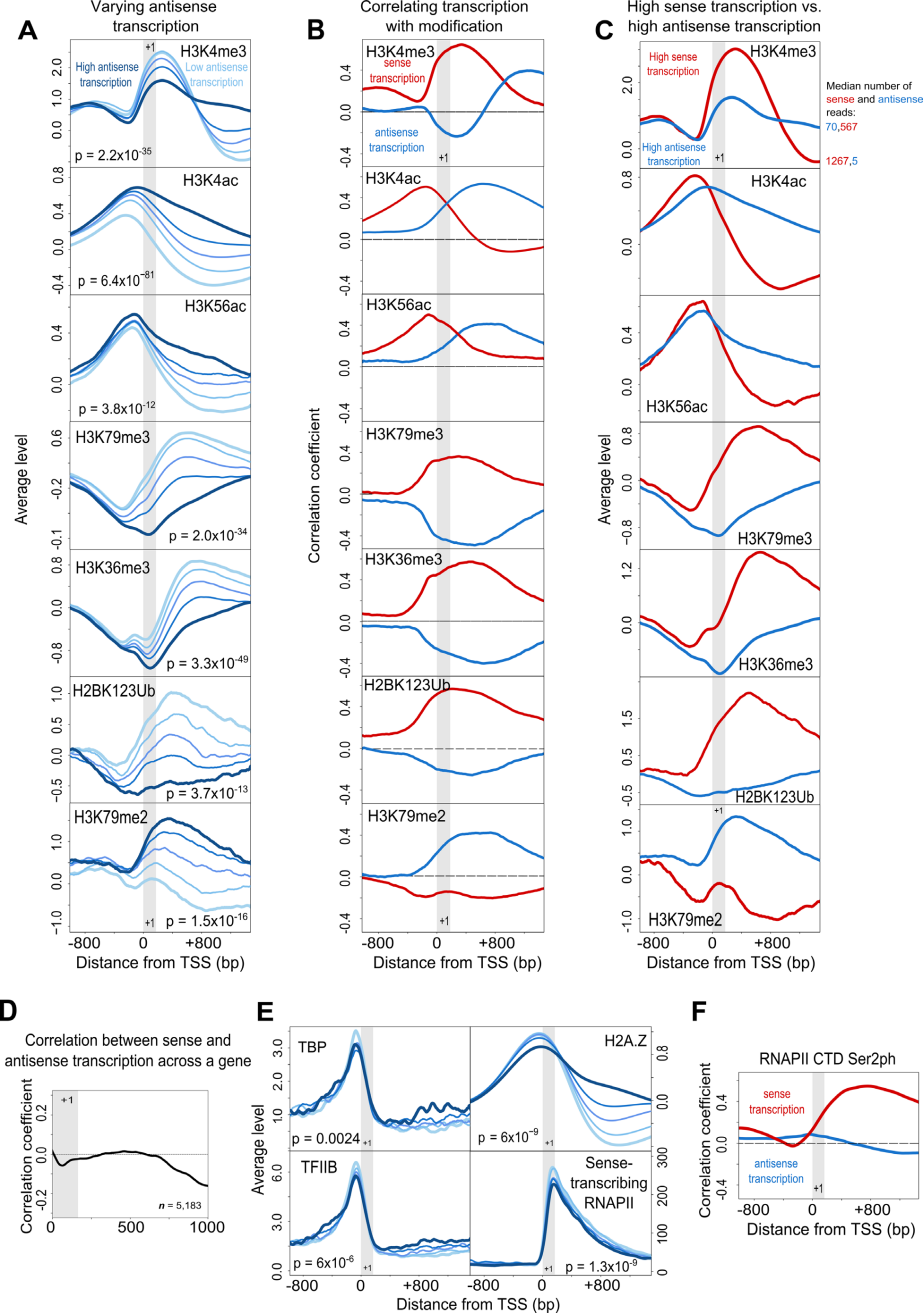
Sense and antisense transcription have distinct associations with a variety of histone modifications. (**A**) The average levels of seven different histone modifications around the TSS (0) in the five gene classes described in Figure 1A, subject to varying antisense transcription. (**B**) The correlation coefficient between the levels of seven different histone modifications in 10 bp windows around the TSS (0) and, separately, the number of sense and antisense NET-seq reads in the 300 bp window described in Figure 1A. (**C**) The average levels of seven different histone modifications around the TSS (0) in the five gene classes described in Figure 1A, subject to high antisense transcription (blue), and high sense transcription (red) in the 300 bp window. (**D**) Spearman correlation coefficient between sense and antisense transcription across genes. (**E**) The average levels of TBP, TFIIB, H2A.Z and sense-transcribing RNAPII. TBP and TFIIB do not spread into the body of the gene with increasing antisense transcription as H2A.Z does. (**F**) Correlation coefficient between level of RNAPII CTD Ser2ph and sense and antisense transcription. Where shown, *P*-values were determined using the Wilcoxon rank sum test, comparing the genes in the highest class to the lowest class near the TSS. See Supplementary Figure S3.

We examined the patterns of histone modifications in genes with varying levels of antisense transcription in the 300 bp window, and identified numerous modifications that showed substantial differences in genes subject to high antisense transcription compared to those with low levels (Figure 3A). These modifications could be broadly classed into three groups: those that were higher across the gene body with high antisense transcription, those that were lower, and those that were more evenly spread. We also assessed how the correlations between chromatin modifications and antisense transcription compared to those correlations between modifications and sense transcription (Figure 3B and C). Genome-wide associations remained the same when the gene class with highest antisense transcription was divided into three subgroups (Supplementary Figure S1) or when regulated TATA-box containing genes (25) were excluded from the analysis (Supplementary Figure S2), indicating a continuum of effects on all gene types, associated with the level of antisense transcription. Although this analysis is based on sense and antisense transcription in the 300 bp window, we show the lack of correlation between sense and antisense transcription extends well into the gene body (Figure 3D). The small negative correlation towards the 3′ region suggests that the initiation of antisense transcription might lead to premature termination of sense transcription, thus affecting steady-state transcript levels.

Acetylation at histone H3 was elevated in genes subject to high antisense transcription (Figure 3A–C; Supplementary Figure S3A–C). Though H3 acetylation was also higher in genes with high sense transcription, these differences were primarily at the promoter, whereas antisense transcription-associated changes occurred both at the promoter and across the gene body.

Levels of H3K4me3 were significantly decreased at the promoter and early transcribed region of genes subject to antisense transcription (Figure 3A–C), but were increased further downstream, suggesting that the mark is redistributed by antisense transcription (31) (Supplementary Figure S3D). Levels of the variant histone Htz1 (H2A.Z) showed a similar redistribution from the promoter into the body of genes subject to high antisense transcription (Figure 3E), i.e. they were more evenly spread. However, TFIIB and TBP remain associated at the promoter, demonstrating that the redistribution we observe is not a result of cryptic promoters in the transcribed region (Figure 3E).

**Figure 4.**
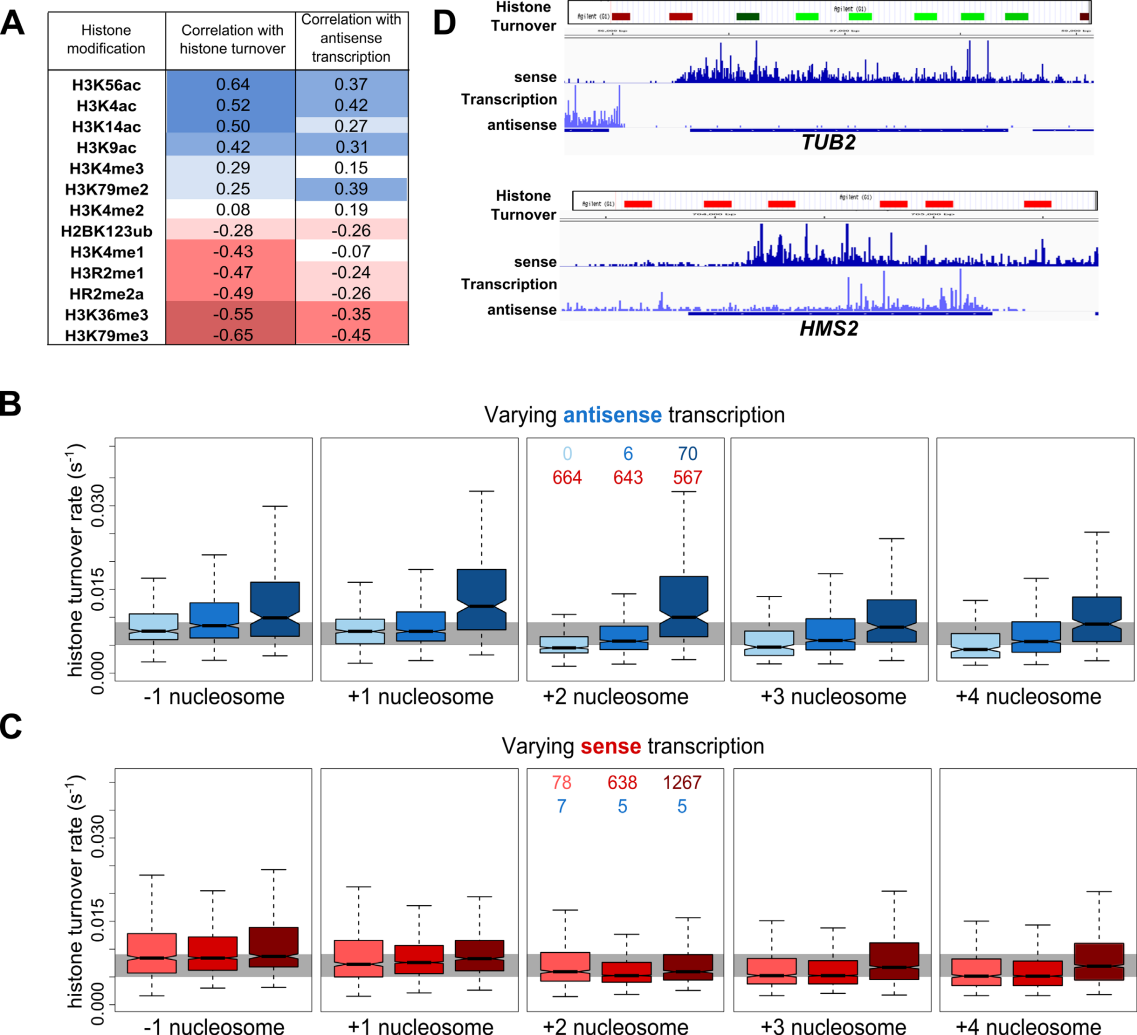
Antisense transcription is associated with increased histone turnover. (**A**) The Spearman's correlation coefficients obtained by correlating histone turnover or antisense transcription genome-wide with the levels of thirteen different histone modifications within the same probed regions. (**B** and **C**) Boxplots showing the distributions of histone turnover rates at the −1 to +4 nucleosomes of genes with (B) varying levels of antisense transcription, coloured as in Figure 1A, with the three intermediate classes combined into a single group, or (C) sense transcription in the 300 bp window. Median levels of sense and antisense transcription in each group are given in red and blue respectively. The top and bottom of the grey box indicates the median value of all probes overlapping a −1 nucleosome or histone turnover genome-wide, respectively. **(D)** Histone turnover data for selected regions in G1 arrested cells from (12) and NET-seq data (8) showing strand-specific transcription for *TUB2* (B) and *HMS2* (C). Green represents regions with low histone turnover and red indicates regions of high histone turnover. *TUB2* and *HMS2* are subject to similar levels of sense transcription but show very different levels of antisense transcription. See Supplementary Figure S4.

H2BK123ub, H3K36me3 and H3K79me3 are lower in genes subject to high antisense transcription genome-wide, mainly across the gene body (Figure 3A–C; Supplementary Figure S3E and F). This is contrary to what one might expect for genes possessing two overlapping, convergent transcription units, given that H3K36me3 is found primarily at the 3′ end of genes (16,19), implying that antisense transcription may be inherently different from sense transcription. Indeed, we observed little correlation between antisense transcription and RNAPII CTD serine 2 phosphorylation (32) (Figure 3F), required for deposition of H3K36me3 by Set2, suggesting that sense and antisense-transcribing RNAPII complexes may themselves differ. Intriguingly, H2BK123ub, H3K36me3 and H3K79me3 are associated with the stabilization of nucleosome structures (16,20–21,33–34). Moreover, despite H3K36me3 not showing a causal relationship with histone turnover (13), the histone modifications most strongly associated with antisense transcription were also those most strongly correlated with histone H3 turnover genome-wide; namely H3K79me3 and H3K36me3 (negatively correlated with both) and H3K4ac, H3K56ac and H3K79me2 (positively correlated with both) (Figure 4A), leading us to ask if antisense transcription correlates with histone turnover.

### Promoters and gene bodies subject to antisense transcription exhibit increased histone turnover rates

To assess histone turnover rates, we utilized a genome-wide map in which the rate of displacement of Myc-tagged H3 by Flag-tagged H3 was measured following induction of Flag-H3 expression (12). Histone turnover was significantly higher between nucleosomes −1 and +4 of genes with high antisense transcription in the 300 bp window compared to those with no/low antisense transcription in the 300 bp window (Figure 4B; *p* = 9.6 × 10^−7^, 1.7 × 10^−22^, 1.9 × 10^−24^, 5.4 × 10^−21^ and 4.9 × 10^−25^ respectively). These trends remained when the H3K36 methyltransferase *SET2* was deleted (16) (Supplementary Figure S4), consistent with no major causal role for H3K36 methylation in the association between antisense transcription and histone turnover.

To confirm that increased histone turnover is a feature of antisense transcription, and not transcription more generally, we assessed how histone turnover differed for genes with varying levels of sense transcription (Figure 4C). Despite large changes in sense transcription between groups, histone turnover did not vary substantially at the promoter, although turnover rates were high. This lack of association between levels of sense transcription and histone turnover at the promoter agrees with previous findings (12). Sense transcription, however, did show the expected relationship at nucleosomes +3 and +4, with higher turnover associated with higher levels of transcription.

The dramatic influence of antisense transcription on histone turnover can be seen at *HMS2* and *TUB2*, genes with similar levels of sense transcription but very different antisense transcription (Figure 4D). Taken together, our results suggest that although histone turnover is a feature of the canonical eukaryotic promoter, it is not correlated with the level of sense transcription, but rather with the level of antisense transcription. This increased turnover could be a consequence of the increased levels of histone chaperones and/or chromatin remodelling enzymes, which have also been shown to direct turnover (35,36).

### Decreasing antisense transcription over *GAL1* increases levels of H3 and H3 acetylation but reduces levels of H3K36me3

**Figure 5.**
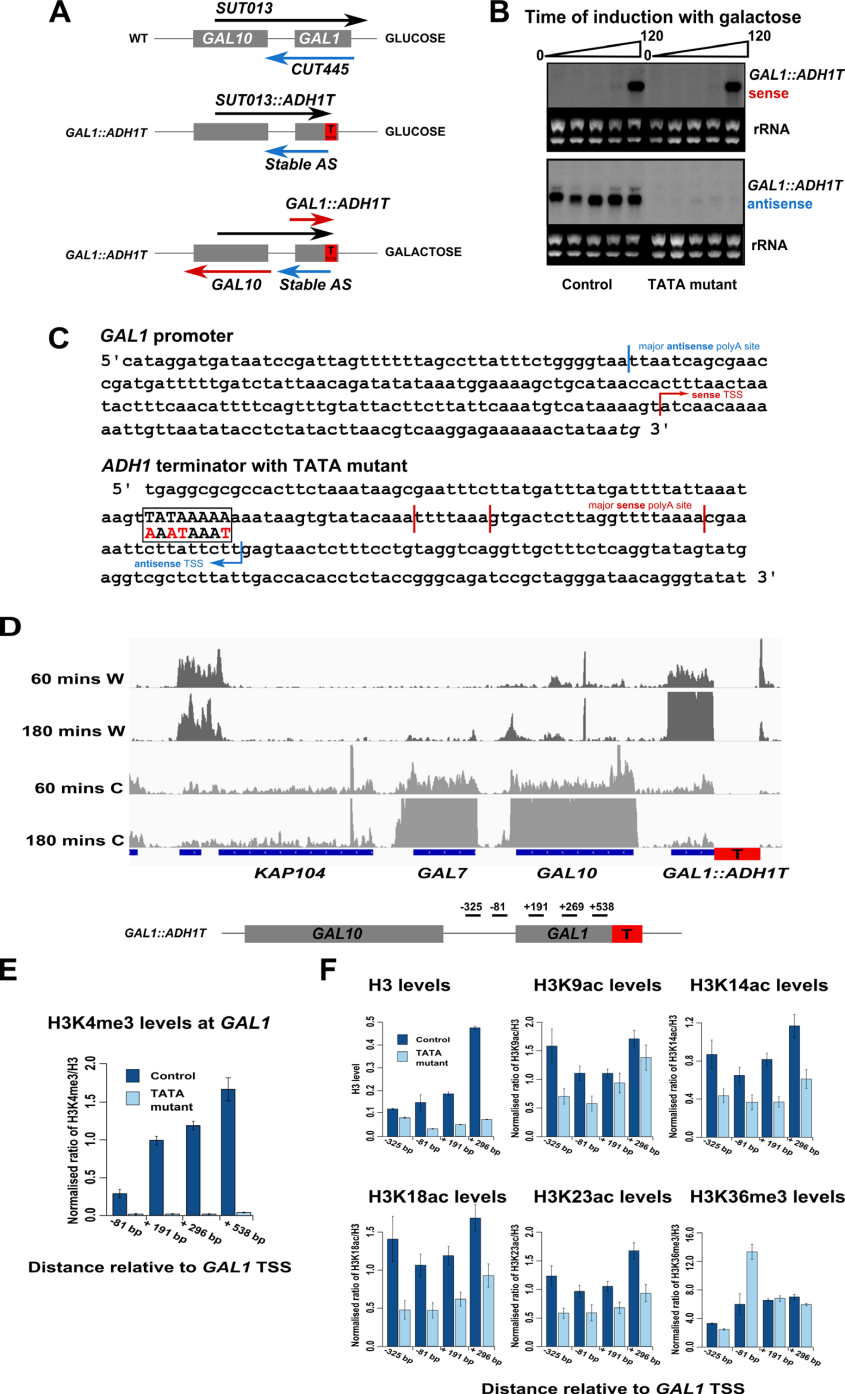
Experimentally varying antisense transcription at *GAL1* alters chromatin modifications (**A**) Schematics showing transcripts at the native GAL locus in glucose medium (top) or after insertion of the *ADH1* terminator (T) +757 bp into *GAL1* in glucose (middle) or galactose (bottom). (**B**) Northern blot probing for the *GAL1* sense and antisense transcripts during an induction time-course (times in min). rRNA in the ethidium bromide-stained gel is shown as a loading control. The control is *GAL1:ADH1_T_* and the TATA mutant has 4 bp of a TATA-like sequence scrambled (see panel C). Antisense transcription into the *GAL1* promoter from the insert can be disrupted by mutation of a TATA-like sequence within *ADH1_T_*. (**C**) *GAL1:ADH1* sense/antisense RNA mapping by RL-PCR (with decapping and dephosphosphorylation) at the 3‘ region of the construct with *ADH1_T_* inserted into *GAL1*. Also shown is the position of the TATA-like sequence, as well as its sequence following mutation (changes marked in red). 3‘ end sites were confirmed using 3‘ RACE. Sequence of primers available on request. (**D**) NET-seq data showing nascent transcripts on the Watson (W) and Crick (C) strand of DNA in a *GAL1:ADH1T* strain after 60 or 180 min in galactose medium. (**E** and **F**) ChIP experiments showing the levels of histone H3 or various histone modifications at *GAL1* in the *GAL1-ADH1_T_* strain, both with and without mutation of the TATA-like sequence. Chromatin was prepared from strains grown in glucose as described previously (27). (E) In the strain with the intact TATA-like sequence, a peak of H3K4me3 is observed corresponding approximately to the site from which the antisense transcript initiates. Following mutation of the TATA-like sequence this peak is lost, consistent with the observed abrogation of antisense transcription, *n* = 2, error bars are SEM. (F) H3 levels, histone lysine acetylation for H3K9, K14, K18 and K23 and histone H3 lysine trimethylation for K36 normalised to H3 levels.

We hypothesised that antisense transcription might itself be responsible for changing histone levels and modifications at the sense promoter and across the gene body, and sought to validate this experimentally at the *GAL1* gene. In both glucose and galactose, *GAL1* has sense and antisense (*CUT445*) transcripts, although in glucose the sense transcript (*SUT013*) originates from a promoter in *GAL10* (22) (Figure 5 and Supplementary Figure S5). Previously, we showed that inserting the terminator sequence of *ADH1* (*ADH1_T_*) into the ORF of *GAL1* resulted in a *redefinition* of the transcription unit; the *GAL1* sense transcripts (*GAL1* and *SUT013*) terminated at *ADH1_T_* while the antisense transcript initiated from it (9) (Figure 5A and B). The shortening of the antisense transcripts during galactose induction results from the production of distinct transcript isoforms (Figure 5B) (8,37). Mapping of these transcripts in glucose revealed that a major antisense transcript end site was 128 bp upstream of the sense TSS, well into the *GAL1–10* promoter, suggesting that it might be able to modulate sense promoter structure (Figure 5C). Mapping nascent transcripts using NET-seq confirmed the presence of antisense transcription extending across the complete *GAL1–10* promoter at the start of induction (60 min in galactose) and when sense transcription is highly induced (180 min in galactose) (Figure 5D).

We identified a sequence within *ADH1_T_* resembling a TATA-box (TATAAAAA) (Figure 5C), and hypothesised that it might be necessary for initiation of the antisense transcript. Strikingly, although downstream of the antisense TSS, mutation of the TATA-box (AAATAAAT) reduced levels of the antisense transcript while sense transcript levels were unaffected (Figure 5B), in keeping with the lack of correlation between sense and antisense transcription (Figure 1C). We used chromatin immunoprecipitation (ChIP) to show reduced levels of H3K4me3 around the TSS for the antisense transcript in the TATA-box mutant strain (Figure 5E), confirming reduced use of the transcription unit. The ability to reduce antisense transcription at *GAL1* provided an experimental system in which the effects of antisense transcription on chromatin could be observed.

The levels of histone and histone modifications were measured using ChIP in glucose-containing medium, specifically H3, a series of H3 acetylation marks (K9,14,18,23ac) and H3K36me3 across both *GAL1* constructs (Figure 5F). ChIP experiments were performed in the presence of *SUT013*, which in glucose is transcribed at similar levels to the *GAL1* antisense transcript (Figure 5D). Based on the genome-wide analysis, it was predicted that H3 and H3 acetylation levels would fall following ablation of antisense transcription, and that H3K36me3 would rise. In agreement with our predictions, levels of H3 and H3 acetylation (normalised to levels of H3) did fall, while levels of H3-normalised H3K36me3 increased, following mutation of the TATA-like sequence and loss of the antisense transcript (Figure 5F). These changes are consistent with a reduced histone turnover/deposition in the absence of antisense transcription, and suggest that antisense transcription is associated with broad changes in chromatin.

### Changes in antisense transcription following deletion of *SET2, RCO1, EAF3* or *SET1* are associated with a corresponding change in nucleosome occupancy and histone modifications

Thus far, we have shown associations between antisense transcription and chromatin features across different genes in a wild-type background. We hypothesised that similar associations would be observed when comparing the *same* genes between *different* yeast strains. i.e. by investigating how antisense transcription changes upon mutation, and whether these changes are mirrored by the changes in the chromatin features discussed above.

**Figure 6.**
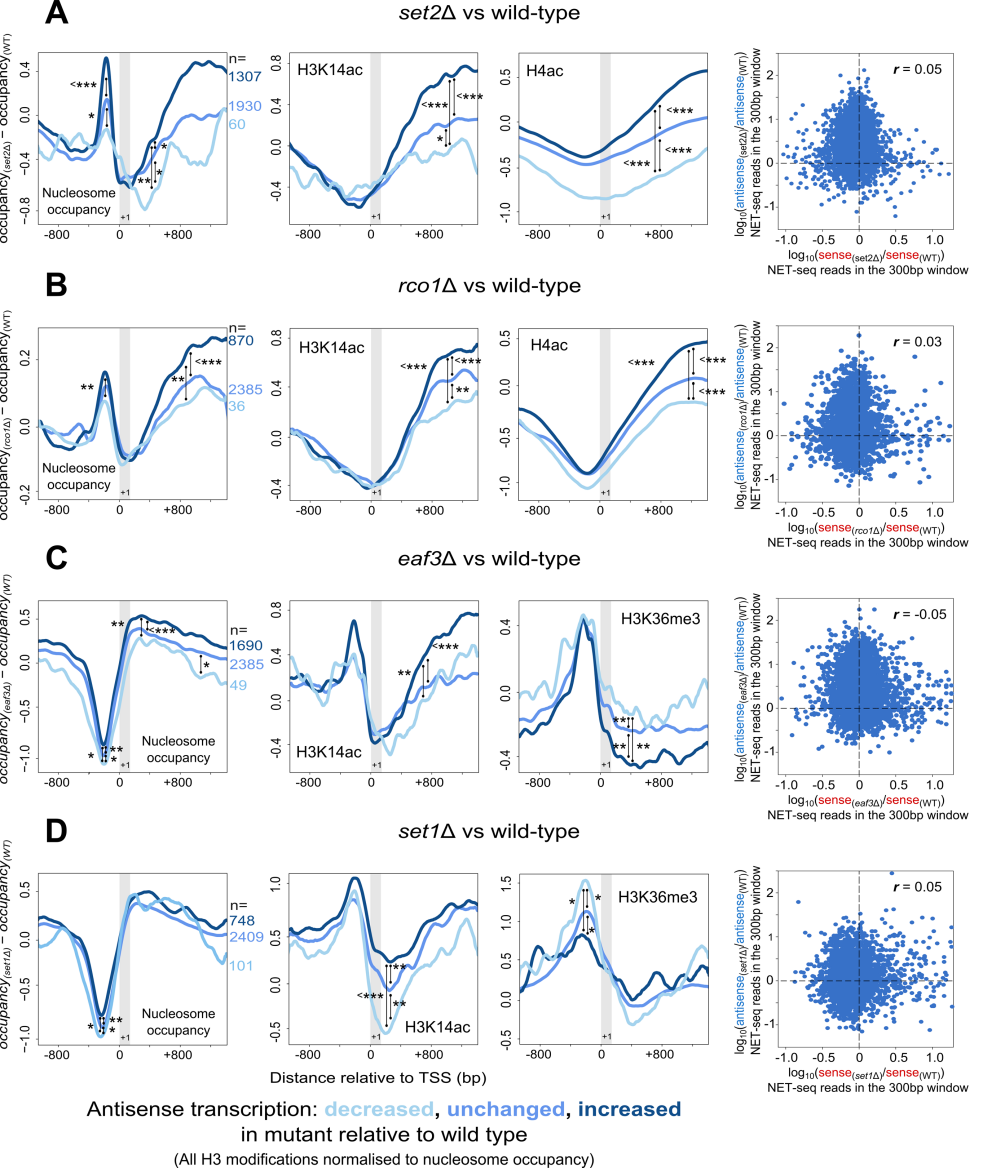
Changes in antisense transcription levels following mutation are concomitant with expected changes in nucleosome occupancy and chromatin modification. (**A**–**D**) The average change in levels of nucleosome occupancy, H3K14ac, H3K36me3 and H4ac in (A) *set2Δ* (B) *rco1Δ* (C) *eaf3Δ* and (D) *set1Δ*, when compared to wild-type. Three gene groups were analysed, which differed in terms of how the level of antisense transcription in the 300 bp window changed following mutation. A positive value indicates the particular level has gone up in the mutant strain relative to wild-type. Black vertical lines indicate *P*-values between gene groups at the points indicated, determined using the Wilcoxon rank sum test (* indicates *P* < 0.05, **P < 0.01, ***P < 0.001). The grey vertical box represents the region typically occupied by the +1 nucleosome. Levels of H3K14ac and H3K36me3 were normalised to levels of nucleosome occupancy as discussed in Materials and Methods. The panels on the far right show scatterplots comparing the changes in sense transcription in the 300 bp window with the changes in antisense transcription for each of the four mutants relative to wild-type. Included are all 5183 genes as discussed in the text. Shown are the Spearman rank correlation coefficients (*r*). NET-seq data were obtained from (7), nucleosome occupancy, H3K14ac and H3K36me3 data were from (38), and H4ac data were from (39).

To this end, we utilised available NET-seq data obtained in yeast mutants known to have altered levels of antisense transcription, namely *SET2*, *RCO1, EAF3* and *SET1* deletion strains (7), and grouped genes on the basis of whether antisense transcription over the 300 bp window increased, decreased or did not change. From the 5183 genes that made up the five classes presented in Figure 1A, we selected three new gene groups based on how they changed classification in the mutant strain compared to wild-type. An ‘increased’ gene group was selected in which genes went up by at least two classes (for example, from class two to class four, in Figure 1A). A second group was selected in which genes dropped by at least two classes (the ‘decreased’ group). The third group contained genes that did not change classification (the ‘unchanged’ group). The numbers of genes in each group for each mutant strain are shown by the left hand panels in Figure 6. Note especially that the three groups are different in each of the four mutants (see corresponding *n* values by the left hand panels), as antisense transcription was differentially altered between them—i.e. a gene classed as ‘increased’ in one mutant could well be classed as ‘decreased’ or ‘unchanged’ in another. Genome-wide levels of sense expression were generally similar between all four mutants and wild-type, save for *eaf3Δ*, in which the average level of sense transcription at a gene was three-quarters that of wild-type. Crucially, and in support of our earlier analyses, the correlation between the change in sense and antisense transcription at the 300 bp window in the deletion mutants relative to wild-type was similarly small for all four strains (Figure 6)—i.e. changes in antisense transcription were not associated with increased or decreased sense transcription.

We considered how levels of nucleosome occupancy and histone modifications compared between these three distinct groups. We obtained levels of nucleosome occupancy, H3K14ac and H3K36me3 from ChIP-seq data (H3K36me3 not shown for *set2Δ* which lacks this modification, or *rco1Δ*) (38). H4ac levels were from ChIP-chip data (available for *set2Δ* and *rco1Δ* strains only) (39). We predicted that genes whose antisense transcription increased following mutation would tend towards a greater increase in nucleosome occupancy and H3K14ac compared to those genes in which antisense transcription decreased, while H3K36me3 would tend towards a greater decrease. The changes in the mutant compared to the wild-type strains are displayed as difference plots (Figure 6A–D). Superimposed on the difference plots for the unchanged gene group are the difference plots for the gene groups with increased or decreased antisense transcription. Strikingly, the changes observed agreed with our earlier analysis (Figures 1–4). For example in the *set2Δ* strain, an increase or decrease in antisense transcription changed nucleosome occupancy at the promoter and in the gene body as predicted. Changes in the levels of antisense transcription were generally found to have reciprocal effects on acetylation and H3K36me3 in the mutant strains (Figure 6D). We note, however, that the changes in chromatin are found in different regions of genes in the different mutant strains. For the *set1* deletion strain, for example, we observed changes in nucleosome occupancy and H3K36me3 at the promoter, but not over the gene body. By contrast, changes in nucleosome occupancy were observed at both the sense promoter *and* the gene body in *set2Δ, rco1Δ* and *eaf3Δ*, while H3K36me3 changed at both regions in *eaf3Δ*. This suggests that Set1 might be required for mediating some of the antisense-transcription associated changes seen at the gene body in the other mutant strains. This analysis supports our hypothesis that antisense transcription is associated with a unique chromatin environment compared to sense transcription. Taken together with the earlier bioinformatics and experimental validation, our data support a model in which increasing antisense transcription levels lead to an increase in nucleosome occupancy and histone acetylation, while decreasing levels of H3K36me3.

## DISCUSSION

**Figure 7.**
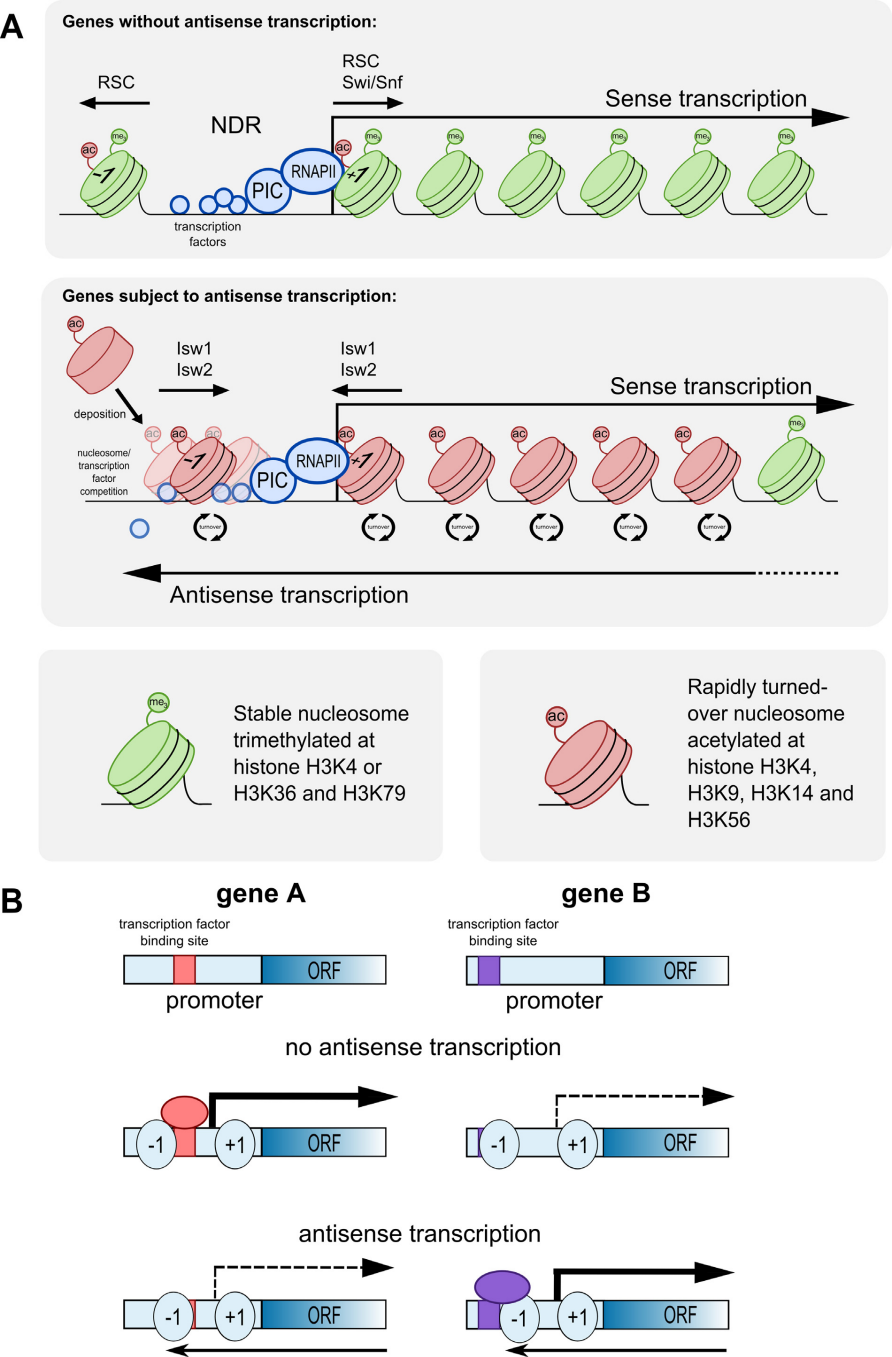
(**A**) Schematic summarising the chromatin dynamics and modifications at a gene subject to sense transcription (top) and one subject to both sense and antisense transcription (bottom). (**B**) Schematic showing how chromatin dynamics resulting from antisense transcription could lead to either activation or repression of two different genes upon loss of antisense transcription. Depending on the initial state of the gene, subtle changes in nucleosome positioning or modification as a result of loss of antisense transcription could facilitate or prevent transcription factor binding and sense transcription. For panels (A) and (B) sense and antisense transcription are not envisaged to be contemporaneous but events in distinct cells in the population (8,23,45).

Here we have shown that genes subject to high levels of antisense transcription show pronounced differences in a broad range of chromatin features, both at the sense promoter and within the gene body. Generally, features associated with newly deposited dynamic chromatin—histone H3 acetylation, turnover, chromatin remodelling enzymes and histone chaperones—are increased, while features associated with established chromatin—H3K79me3, H3K36me3 and H2B123ub—are reduced. These are distinct from the features associated with sense transcription (Figure 7A). Moreover, levels of sense and antisense transcription are not correlated with one another genome-wide. That nascent antisense transcription is relatively abundant, and that it has such pronounced associations with chromatin in a manner distinct from sense transcription, suggests that it should be considered an important and canonical regulatory feature of yeast genes.

The lack of correlation between sense and antisense *transcription* are somewhat at odds with the current perception of the field supporting an antagonistic relationship between sense and antisense *transcripts*, based on single gene studies (22–24,40). Given the lack of correlation between nascent sense and antisense transcription described in this work, we expect negative regulation will exist at some genes, while positive relationships will exist at others, as reported for some sense and antisense transcripts (41,42). Indeed, tiling array analysis of sense and antisense transcript levels in yeast undergoing environmental change reveals that antisense transcripts show both reciprocal and positive relationships with sense transcripts (43,44), consistent with our data showing no correlation between changes in sense and antisense transcription following a change in environment (Figure 1D). We also expect some genes to show no change upon loss of an antisense transcript, as we observe for *GAL1*. Thus changes to antisense transcription do not necessarily influence overall levels of sense transcription, though they may influence the number of cells in the population expressing sense. This is illustrated by *PHO84* where low levels of antisense transcription are sufficient to bring about changes in chromatin, influencing the number of cells expressing the sense transcript (45). *PHO84* has a nucleosome occupied promoter, predicted by our data to be a function of antisense transcription. This apparent paradox between high levels of sense transcription and an antisense-related nucleosome occupied promoter is explained by the mutually exclusive and skewed distribution of sense or antisense transcripts in a population of cells. Sense transcripts, at very high levels, are present in only one in five cells in the population (45). This proportion of cells with sense transcript is reduced further in mutants with increased levels of antisense transcript, but the number of sense transcripts per cell remains similar. The remaining cells have an antisense transcript or neither transcript. Thus in four out of five cells in the population, the −1 and +1 nucleosomes at the *PHO84* promoter are likely to be in the closed chromatin configuration, with only one in five cells showing a sense-specific open promoter chromatin configuration. These data suggest that low levels of antisense transcription should not be dismissed as irrelevant and that single cell experiments can reveal changes in the distribution of transcripts in a population, influenced by levels of antisense transcription.

At *GAL1*, we show that reducing levels of antisense transcripts leads to expected changes in histone features, including increased H3 acetylation but reduced H3K36me3. However, there is a notable absence of changing histone modifying enzyme levels between the antisense transcription classes that might explain these differences. Of the factors in Table 1, only two are associated with histone modifying activities, Spt3 and Rpd3. Spt3 is a negative regulator of the Gcn5 lysine acetyltransferase (46) while Rpd3, best known as a histone deacetylase (HDAC), also has histone chaperone activity (47). Spt3 and Rpd3 show similar genome-wide correlations with chromatin to Isw1 and Ino80 but distinct from Sin3, a component of the Rpd3 HDAC complexes, notably nucleosome occupancy at −1 (*r*_s_ = +0.221, +0.186, +0.301, +0.257, −0.246, respectively), H3K9ac (*r*_s_ = +0.283, +0.245, +0.242, +0.257, −0.049) and H3K18ac (*r*_s_ = +0.202, +0.197, +0.230, +0.236, −0.154), suggesting that Rpd3 has an HDAC complex- independent function related to antisense transcription. Furthermore, although histone modifications, antisense transcription and histone turnover are strongly correlated with each other, histone modification is not thought to cause changes in histone turnover, nor is histone turnover thought to change the histone modifications (13). We suggest that antisense transcription might represent the missing link that causes histone turnover, although this has not yet been demonstrated experimentally.

Some regulatory non-coding transcripts, including *SUT013* at the GAL locus, are reported to deposit H3K36me3 during transcription to repress gene transcription (22–24), while our data suggest that antisense transcription reduces H3K36me3. In contrast to sense transcription, we show that serine 2 phosphorylation of the CTD, required for Set2-dependent H3K36me3 (48) does not increase over antisense transcription units, explaining reduced H3K36me3. Thus, the difference between different types of non-coding transcription events may reflect the way the transcript are initiated and the patterns of CTD phosphorylation on elongating RNA polymerase II. Given that ablation of *SUT013* gives rise to increased levels of various non-coding transcripts reading antisense from *GAL1* into *GAL10* (Supplementary Figure S5), an alternative explanation might be that these new transcripts are responsible for the reduced levels of H3K36me3 and increase acetylation. This observation illustrates one of the potential difficulties of working with non-coding transcripts/transcription—that manipulations of the genome to remove one transcript often give rise to novel spurious transcripts such as described here at the *GAL* locus or at the *HMS2* locus (9).

How might the observed changes to chromatin affect gene behaviour? The promoters of genes with antisense transcription also share common dynamic chromatin features with the promoters of genes that are highly *plastic*—i.e. genes with the capacity to be extensively regulated. Transcriptionally plastic genes possess occupied promoters, have higher histone turnover rates and are more extensively regulated by chromatin remodelling enzymes (49). Antisense transcripts have already been implicated in transcriptional plasticity (43) but, until now, the association between antisense transcription and these chromatin features has not been evident. How might antisense transcription enhance plasticity in this way? One possible mechanism is that antisense transcription could increase the potential number of chromatin configurations permissible at a promoter, by, for example, recruiting chromatin remodelling enzymes. More possible configurations would mean more potential combinations of bound transcription factors. This could in turn increase the variety of transcription complexes that can form across different environments, and so enhance plasticity. In support of this, genes with more transcription factor binding sites—i.e. those with inherently more possible permutations of bound and unbound sites—are more transcriptionally plastic (50). The association between antisense transcription and NDR size could provide an explanation as to why antisense transcription can be both activating and repressive under different genomic contexts—closing of the NDR could either occlude or unveil binding sites involved in stabilisation of the transcription complex (Figure 7B).

Genes that show marked variation in expression levels within a population of genetically identical cells are described as noisy (51,52). Strikingly, the chromatin features we find associated with antisense transcription—a closed NDR at the 5′ sense promoter, increased nucleosome occupancy and H3 acetylation, and decreased H3K79me3, H3K36me3 and H2BK123 ubiquitination—are also associated with gene expression noise (49,53). It is possible, therefore, that by conferring these chromatin changes, antisense transcription is itself acting as a noise generating mechanism. Crucially, gene expression noise can be altered *without* changing the overall level of expression itself, thus antisense transcription could raise noise without changing the level of sense transcription, in line with the lack of correlation observed between the two processes. Intrinsically noisy genes are subject to a distinct mode of transcription initiation in which a gene switches between an off state and an active, rapidly re-initiating state, referred to as transcription *bursting* (53,54). Antisense transcription could favour bursting by NDR closure, which might make the promoter more bistable and therefore stochastic in nature, forcing it to switch between rapid bursts of transcription and chromatin repression (55). Use of RNA-FISH might provide a means of identifying whether antisense transcription can enhance expression noise.

## Supplementary Material

SUPPLEMENTARY DATA
