# Supplementary material for: Sense and antisense transcription are associated with distinct chromatin architectures across genes
Source: Nucleic Acids Res. 2015 Jun 29;43(16):7823–37. doi: 10.1093/nar/gkv666 (PMC4652749; doi:10.1093/nar/gkv666)
Supplement: SUPPLEMENTARY DATA [file supp_43_16_7823__index.html]

Sense and antisense transcription are associated with distinct chromatin architectures across genes — SUPPLEMENTARY DATA 

# Sense and antisense transcription are associated with distinct chromatin architectures across genes

## SUPPLEMENTARY DATA

- SUPPLEMENTARY DATA
