# Supplementary material for: Sense and antisense transcription are associated with distinct chromatin architectures across genes
Source: Nucleic Acids Res. 2015 Jun 29;43(16):7823–37. doi: 10.1093/nar/gkv666 (PMC4652749; doi:10.1093/nar/gkv666)
Supplement: SUPPLEMENTARY DATA [file supp_gkv666_nar-00577-x-2015-File009.pdf]

Sense and antisense transcription are associated with distinct chromatin architectures across genes

Struan C. Murray, Simon Haenni, Françoise S. Howe, Harry Fischl, Karolina Chocian, Anitha Nair, Jane Mellor[1]*.

Table S1 associated with Table 1: Factors enriched (up) or depleted (down) at the promoters of high antisense genes versus low antisense genes. Factors shown are those deemed significantly enriched or depleted ($p < 0.00001$). The two gene groups considered were the highest and lowest antisense transcribed groups shown in Fig. 1A.

FACTOR	COMPLEX	level in low antisense genes	level in high antisense genes	Change in level	p-value	Reference
Isw1	ISW1a, ISW1b	0.38678125	0.636591207	up	4.22E-09	(1)
Ino80	INO80.COM	0.487884342	0.765456466	up	4.85E-09	(2)
Pob3	FACT	0.212806998	0.36593989	up	2.46E-07	(3)
Rsc9	RSC1, RSC2, RSCa	0.395981022	0.603001709	up	3.19E-07	(4)
Swi3	SWI-SNF	0.356198807	0.594433251	up	4.85E-07	(5)
Sir3	SIR	-0.099840876	-0.25654192	down	1.14E-06	(6)
Rpt6	APIS	-0.274200788	-0.467605783	down	2.10E-06	(7)
Rpd3	RPD3	0.4482388	0.645696734	up	2.91E-06	(8)
Rpb7	Pol II	0.268829928	0.538684777	up	3.77E-06	(9)
Itc1	ISWI	0.519809341	0.695099945	up	1.21E-05	(1)
Spt3	SAGA, SLIK	0.498519002	0.705706159	up	1.91E-05	(10)
Ctk1	CTK	0.447113506	0.632521988	up	2.07E-05	(11)
Iws1	SPT6 interactor	-0.12502432	0.01829391	up	2.23E-05	(3)
Spt6	SPT6	-0.069800858	0.065470059	up	2.86E-05	(3)
Rpo21	Pol II	-0.006376794	0.187252235	up	3.79E-05	(9)
Htb2	nucleosome	0.108396772	0.225449777	up	6.68E-05	(12)
Rpb2	Pol II	-0.045057778	0.183859738	up	8.78E-05	(9)
Bur6	NC2	0.499996936	0.343628513	down	8.92E-05	(13)
Jhd1	JHDM1	-0.179841782	-0.29733578	down	9.80E-05	(14)

Table S2 associated with Table 1: Factors enriched (up) or depleted (down) at the promoters of highly antisense transcribed genes versus sense transcribed genes. Factors shown are those deemed significantly enriched or depleted ($p < 0.00001$). Sense transcribed genes were selected as those which had no antisense transcription and a median read count of 948 for sense transcription. High antisense transcribed genes had a median read count of 70 for the antisense strand and 578 for the sense strand.

FACTOR	COMPLEX	level in high sense genes	level in high antisense genes	Change in level	p-value
Ssl1	TFIIH	1.603591834	0.98704757	down	1.29E-14
Vps72	SWR-C	0.845451917	0.465502855	down	2.18E-10
Rad3	TFIIH, NER3	1.346104517	0.856531532	down	1.52E-09
Swc1	SWR-C	0.630525628	0.306542191	down	2.78E-09
Bur6	NC2	0.693121705	0.310860265	down	4.26E-09
Isw1	ISW1a, ISW1b	0.398116866	0.759194565	up	1.77E-08
Esa1	NuA4/Piccolo	0.15106436	-0.041755201	down	6.22E-08
Taf13	TFIID	0.4253047	0.174488633	down	1.55E-07
Snf1	SNF1	0.231795628	0.011201899	down	2.60E-07
Tfb1	TFIIH	0.71225726	0.416035268	down	4.97E-07
Ino80	INO80.COM	0.508630658	0.848911861	up	9.21E-07
Pob3	FACT	0.249384191	0.460868601	up	9.96E-07
Ncb2	NC2	0.35900391	0.139012264	down	1.62E-06
Rpt6	APIS	-0.227445708	-0.53365289	down	2.38E-06
Sir3	SIR	-0.060353652	-0.305839911	down	3.53E-06
Kin28	TFIIH	-0.028116073	-0.419671378	down	1.24E-05
Htz1	nucleosome	1.205801394	0.822129715	down	1.48E-05
Mot1	Mot1	0.173703676	0.005442068	down	3.17E-05
Tfb3	TFIIH	0.413703033	0.196812728	down	3.87E-05
Itc1	ISWI	0.51342015	0.745109255	up	6.93E-05

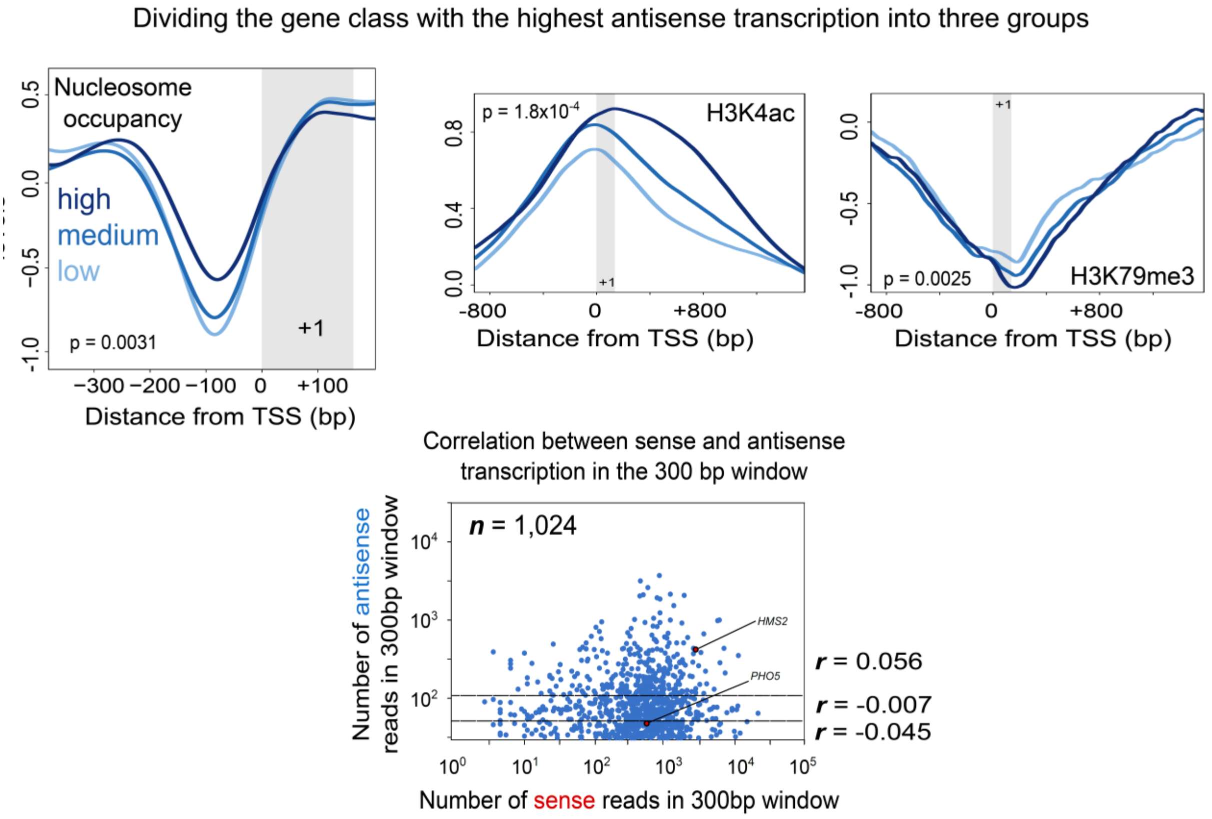

Fig S1 (associated with figures 1-3). Trends associated with antisense transcription remain when only genes in the highest antisense transcription class (see figure 1A) are subdivided into three equal groups. Shown are the average levels of nucleosome occupancy (**top-left**), H3K4ac (**top-middle**) and H3K79me3 (**top-right**) in the vicinity of the TSS, for three subclasses of genes drawn from the class with the most antisense transcription, equally subdivided according to the level of antisense transcription in the 300bp window. P-values shown are obtained by comparing distributions at the minimum point for nucleosome occupancy and the maximum for H3K4ac and H3K79me3 (Wilcoxon rank sum test). (**bottom**) Scatter plot of the genes in these three subclasses, demonstrating that the correlation between sense and antisense transcription remains very small in these groups (-0.045,-0.007 and 0.056 for the low, medium and high class respectively). Correlation coefficients (*r*) given are Spearman rank coefficients.

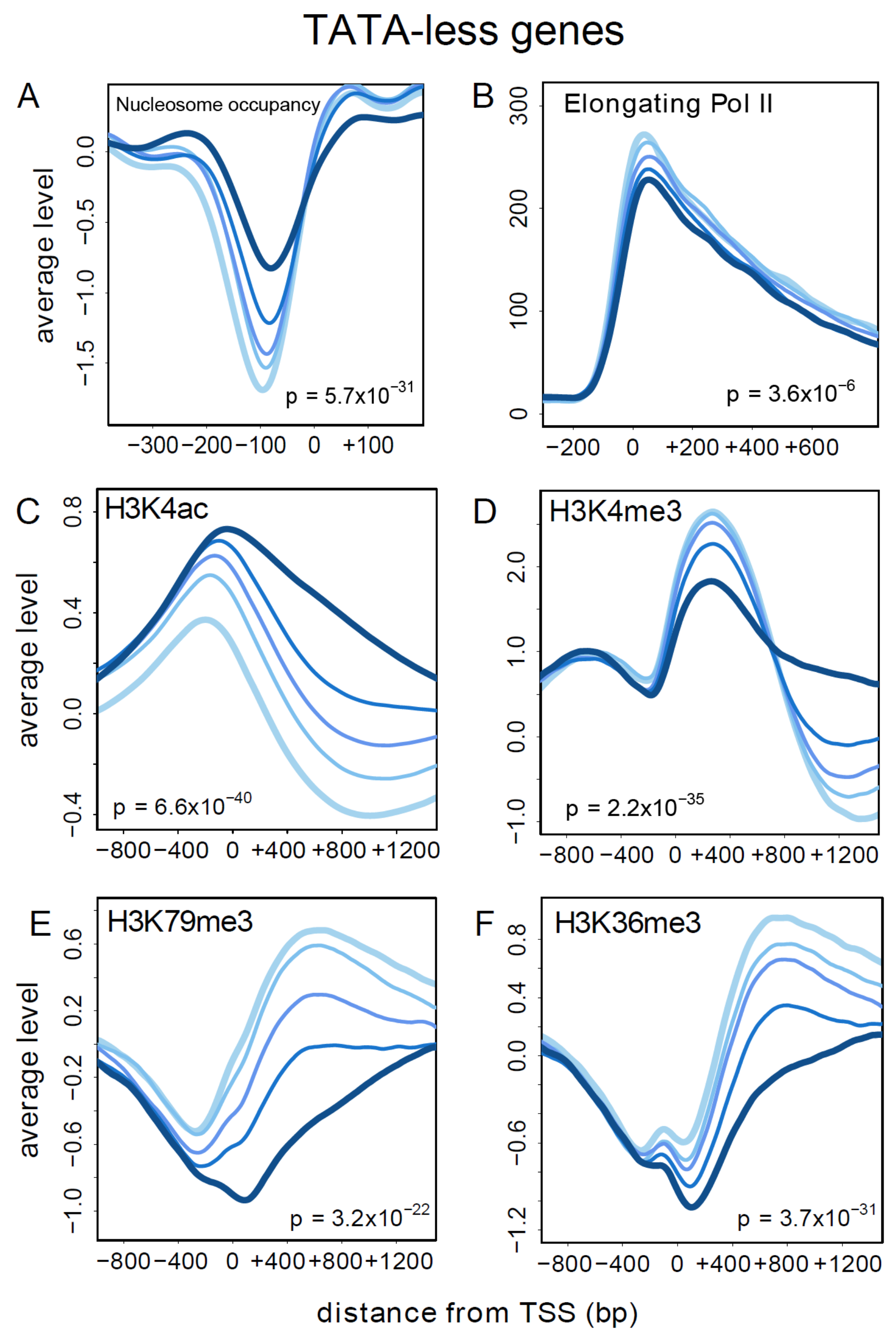

Figure S2 (associated with figures 1-3): Trends associated with antisense transcription remain when genes with TATA-boxes are excluded from the analyses. Shown are the average levels of (**A**) nucleosome occupancy, (**B**) sense-transcribing Pol II, (**C**) H3K4ac, (**D**) H3K4me3, (**E**) H3K79me3 and (**F**) H3K36me3 in the vicinity of the TSS of TATA-less (15,16) genes with varying levels of antisense, as defined in the main text and Figure 1**A**. This analysis suggests that although genes with TATA boxes generally have higher levels of antisense transcription than genes without TATA boxes, the associations between chromatin and antisense transcription are the same at TATA-less promoters as TATA-containing. We conclude that antisense transcription affects all classes of yeast gene and is not specific for SAGA-regulated, TATA-box genes.

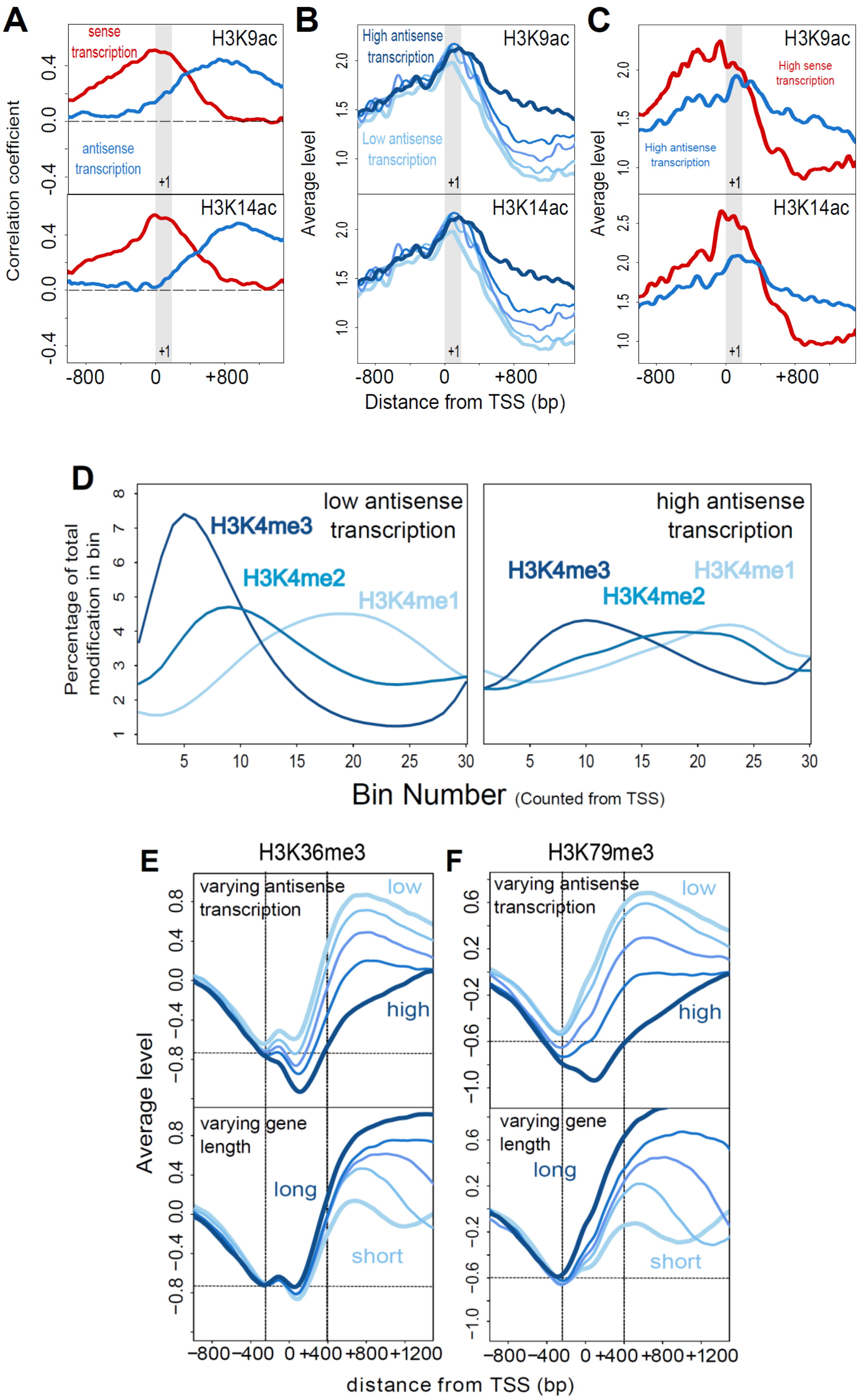
A
sense transcription
H3K9ac
antisense transcription
H3K14ac
Correlation coefficient
B
High antisense transcription
Low antisense transcription
H3K9ac
H3K14ac
Average level
Distance from TSS (bp)
C
H3K9ac
High sense transcription
High antisense transcription
H3K14ac
Average level
+1
-800
0
+800
D
low antisense transcription
high antisense transcription
H3K4me3
H3K4me2
H3K4me1
Percentage of total modification in bin
Bin Number (Counted from TSS)
E
H3K36me3
F
H3K79me3
varying antisense transcription
low
high
varying gene length
long
short
Average level
distance from TSS (bp)
−800−400 0 +400 +800 +1200

Figure S3 (associated with figure 3): Genome-wide associations between antisense transcription and histone modification. (A-C) Antisense transcription is associated with a rise in H3K9ac and H3K14ac across the gene body. (**A**) The correlation coefficient between the levels of H3K9ac and H3K14ac in 10bp windows around the TSS (0) and, separately, the number of sense and antisense NET-seq reads in the 300bp window described in Fig. 1A. (**B-C**) The average levels of H3K9ac and H3K14ac around the TSS (0) in the five gene classes described in Fig. 1**A**, subject to (**B**) varying antisense transcription and (**C**) high antisense transcription (blue), and high sense transcription (red) in the 300bp window. H3K9ac and H3K14ac show a similar relationship with antisense transcription compared to H3K4ac – antisense transcription is associated with an increase of H3K4ac at the promoter and, to a greater extent, the +1 nucleosome and remainder of the gene body. (**D**) **Genes with high antisense transcription show more evenly distributed H3K4 methylation profiles**. The distribution of H3K4me1, H3K4me2 and H3K4me3 within genes with both low and high levels of antisense transcription (left and right panels respectively). Genes were divided into the 30 bins and the level of modification determined as a percentage of the total modification across the gene. The peak of H3K4me3 normally found at the beginning of the transcription unit is much less pronounced in genes with high antisense transcription, in line with previous observations on single genes (17,18). One explanation for the more even distribution of H3K4 methylation marks over genes with high antisense transcription is that it is the result of having two, overlapping, and opposing transcription units. This is in line with the rise in H3K4me3 seen in the gene body of genes with high antisense transcription, a possible consequence of 3’ promoters directing antisense transcription. (**E-F**) **The association of antisense transcription with decreased H3K36me3 and H3K79me3 is not related to gene length.** The association of antisense transcription with decreased H3K36me3 (**E**) and H3K79me3 (**F**) at the promoters of genes is independent of the known association between gene length and these marks. Shown is the average level for both marks at varying gene length (divided into groups of approximately 500, 1000, 1500, 2000 and 2500bp) compared to the average levels in genes with varying levels of antisense. Horizontal dotted black lines correspond to the minimum average value found in genes of varying length. Vertical dotted black lines have been added to illustrate a region approximately encapsulating the promoter and the -1, +1 and +2 nucleosomes. Neither H3K79me3 nor H3K79me3 show substantial changes in this region when length is varied, but do when the level of antisense is varied.

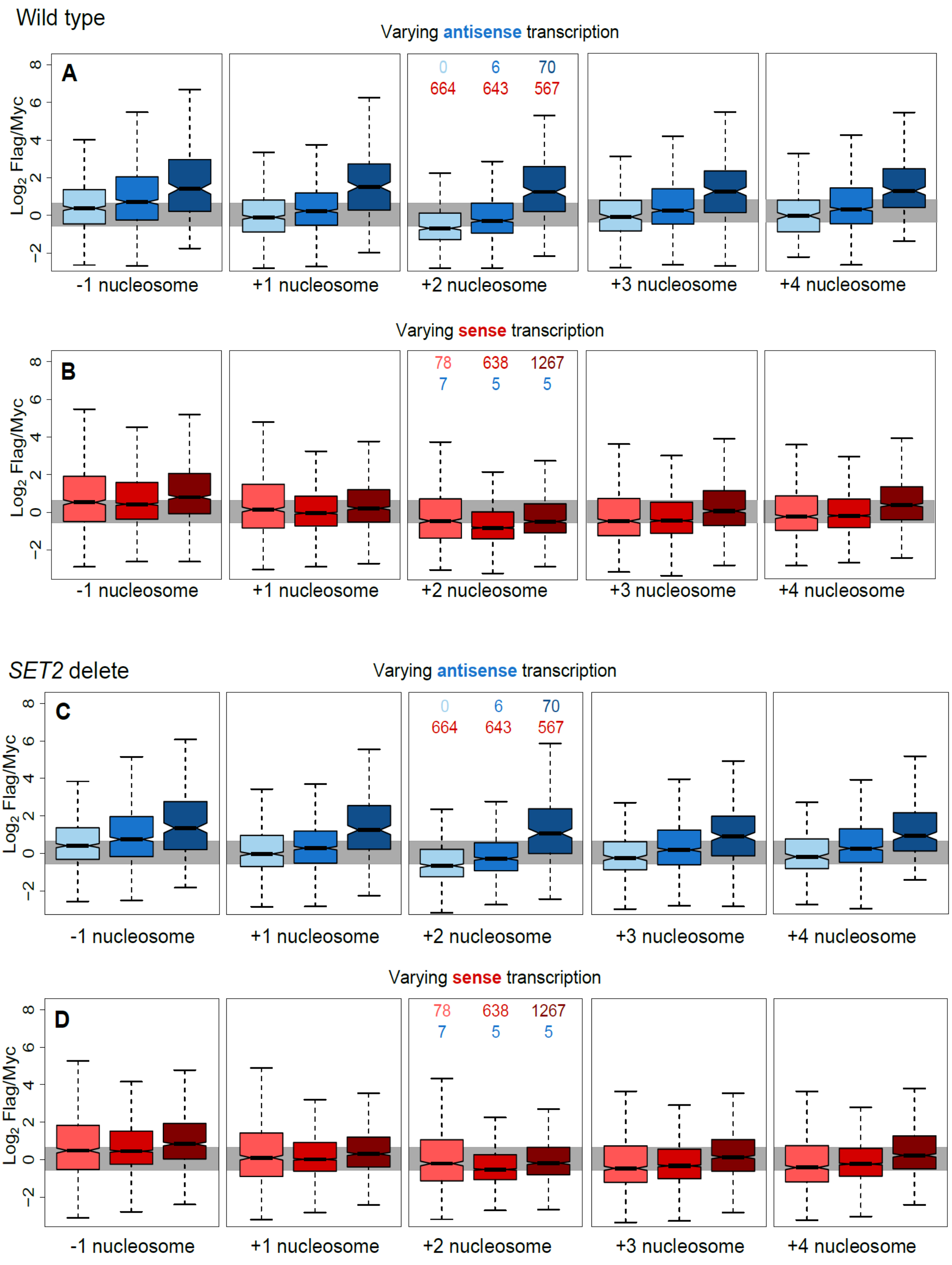

Wild type
Varying antisense transcription
A
Log2 Flag/Myc
0 6 70
664 643 567
-1 nucleosome
+1 nucleosome
+2 nucleosome
+3 nucleosome
+4 nucleosome
Varying sense transcription
B
78 638 1267
7 5 5
SET2 delete
Varying antisense transcription
C
0 6 70
664 643 567
Varying sense transcription
D
78 638 1267
7 5 5

Figure S4 (associated with Fig. 4): Histone turnover as assessed using an alternative genome wide data set from Venkatesh et al., (19) in a wild-type strain background (A-B) and a strain in which *SET2* was deleted (C-D). (**A**) Boxplots showing the distributions of the log2 Flag/Myc ratio (an estimate of histone turnover rate) at the -1 to +4 nucleosomes of genes with varying levels of antisense transcription, specifically the five classes defined in Figure 1B, with the three intermediate classes combined into a single group. Median values of sense and antisense transcription in each group are given in red and blue respectively. The bottom of the grey box indicates the median value of histone turnover genome-wide, while the top of the box indicates the median value of all probes overlapping a -1 nucleosome. (**B**) Boxplots of histone turnover at the -1 to +4 nucleosomes, with the genes divided on the basis of sense transcription in the 300bp window shown in Figure 1**A** (**C**) Boxplots showing the distributions of the log2 Flag/Myc ratio (an estimate of histone turnover rate) at the -1 to +4 nucleosomes of genes with varying levels of antisense transcription, specifically the five classes defined in Figure 1B, with the three intermediate classes combined into a single group. Median values of sense and antisense transcription in each group are given in red and blue respectively. The bottom of the grey box indicates the median value of histone turnover genome-wide, while the top of the box indicates the median value of all probes overlapping a -1 nucleosome. (**D**) Boxplots of histone turnover at the -1 to +4 nucleosomes, with the genes divided on the basis of sense transcription in the 300bp window shown in Figure 1**A**.

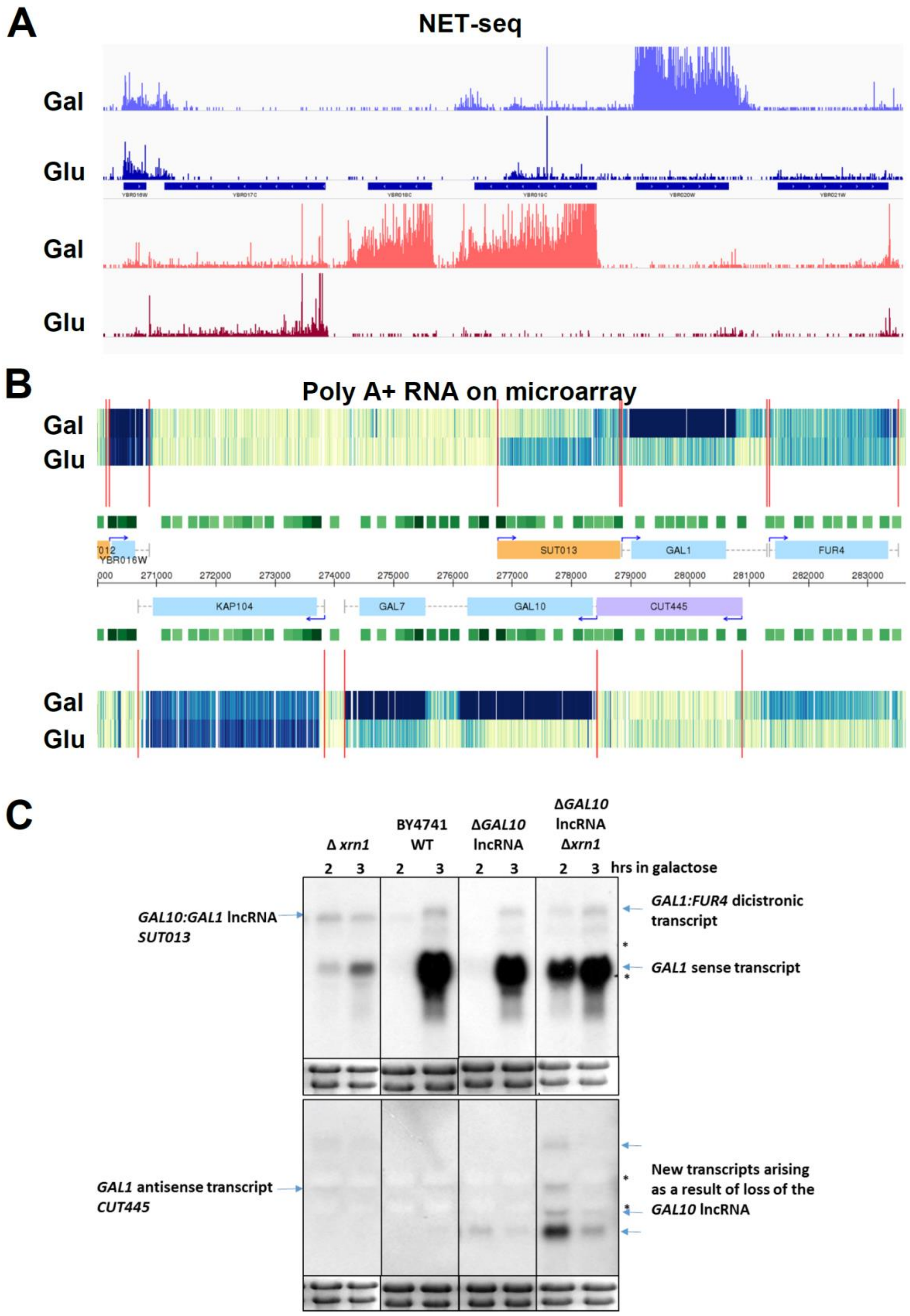

Fig S5 (associated with figure 5). Characterisation of transcription and transcripts at the GAL locus. (**A**) NET-seq showing nascent transcription at the GAL locus in cells cultured in glucose (Glu) or after 3 hours in galactose (Gal) visualised in the IGV viewer (20). The Watson strand

is shown above the gene map and the Crick strand below the map. The scale for the read counts is 0-20 and each read represents a single 3'OH associated with RNA polymerase II. (**B**) Strand specific microarray hybridized with poly A+ RNA prepared from cells cultured in glucose (Glu) or after 3 hours in galactose (Gal). The darker blue the signal, the more transcript is present. Quantitative data for the NET-seq and microarray are available from Nguyen et al (2014) (21). (**C**) Autoradiograph of a Northern blots showing transcripts at *GAL1* in the strains indicted after 2 or 3 hours induction in YP galactose. The northern blot was hybridized to strand-specific probes, identical to those used in figure 5, labelled to similar specific activities. The blots for the sense (top) and antisense (bottom) probes were exposed to film for the same time. * mark the position of the ribosomal RNAs also shown below each panel by way of a loading control. The blue arrows mark the positions of transcripts of interest. Strain lacking the 5' to 3' exonuclease Xrn1 were used to reveal the Xrn1-sensitive transcripts. These include *SUT013* and all the antisense transcripts. Mapping of the stable antisense transcript in the Δ*GAL10* lncRNA strain revealed a major transcription initiation site at +667 with respect to the *GAL1* ATG. Transcripts extend over the *GAL10-1* promoter and into the *GAL10* coding region. Total levels of *GAL1* antisense transcripts in the Δ*GAL10* lncRNA Δ*xrn1* are qualitatively higher than levels of *SUT013* in the *xrn1*Δ. This data is included to provide an explanation for why loss of the *GAL10-1* lncRNA (*SUT013*) (strain Δ*GAL10* lncRNA) results in loss of K36me3 and increased H3ac (22). We predict that increased *GAL1* antisense transcription resulting from loss of *SUT013* transcription would lead to these chromatin changes, consistent with our model.

Supplementary Methods

Genome-wide data Genome-wide levels of Spt15 (TBP) and Sua7 (TFIIB) were obtained from Venters and Pugh (23) (E-MEXP-1677). Levels of H3K36me3, H3K79me3, H3R2me and H3R2me2a were obtained from Kirmizis et al., (24)(GSE14453). Levels of H3K4me1, H3K4me2 and H3K4me3 were obtained from Kirmizis et al.,(18) (GSE8626). Levels of H3K4ac were obtained from Guillemette et al., (25)(GSE27307). Levels of H3K56ac were obtained from Xu et al., (26) (E-MEXP-1248). Levels of H3K9ac and H3K14ac were obtained from Pokholok et al., (17)(E-WMIT-3). Levels of H3K79me2 and H2B normalised levels of H2BK123 ubiquitination were obtained from Schulze et al., (27) (E-MEXP-3217). Levels of Htz1 were obtained from Guillemette et al., (28). RNAPII CTD Ser2 phosphorylation data was obtained from Kim et al., (14). Levels of H4ac or H3K36me3, H3K14ac and nucleosome occupancy were obtained from Pokholok et al., (17) or Chabbert et al., (29).

Antibodies for ChIP. Antibodies were obtained from Abcam: H3K36me3 ab9050 or Millipore: H3K9ac 07-352, H3K14ac 07-353, H3K18ac 07-354, H3K23ac 07-355, H3K4me3 05-747R, H3 07-690.

Genome wide correlations with nascent transcription To determine the level of association between histone modifications and both sense and antisense-transcribing Pol II, the yeast genome was first divided into overlapping 50bp windows. For those windows that fell within a single transcription unit (i.e. that did not lie outside transcription units or within a region overlapped by two transcription units with reverse orientation), the level of nascent sense and antisense transcription was determined, as was the level of numerous histone modifications. A Spearman rank correlation coefficient was then determined for each of these modifications by correlating levels of the modification with the level of nascent sense and, separately, antisense transcription in the same window.

Transcription machinery at promoters The occupancies of 202 different transcription-related proteins at gene promoters were determined using a comprehensive ChIP-chip study in TAP-tagged yeast strains as discussed in the main text. P-values were determined using the Wilcoxon rank-sum test and comparing the distribution of occupancy levels between two gene groups. Data shown in the paper are those for factor occupancies obtained at 25˚C within a 60 bp region between -320 and -260 relative to the start codon (on average -240 to -180 relative to the TSS). The analysis was repeated using data obtained at the -90 to -30 region relative to the start codon (on average -10 to + 50 relative to the TSS), and gave similar results (data not shown). Similar results were also obtained using occupancy data obtained at 37˚C (data not shown), suggesting the results are robust within this temperature range. The high and low antisense transcription groups used were the top and bottom gene classes shown in Fig 1A (n = 1024 and 1240 respectively). Data were not available for all genes; for the 202 factors the percentage of genes for which data were available averaged at 71%, with a standard deviation of 19%. Values for a given gene were not discarded if they did not exceed the 5% FDR cut-off, as we did not want to exclude genes at which there was only low level (or no) binding.

H3K4 methylation distributions To assess the distribution of H3K4 methylation across genes the gene body was divided into 30 bins, and the level in each bin was determined as a fraction of the total level across the entire gene body.

Mapping of transcript initiation and termination sites Initiation and termination sites for both sense and antisense transcripts at *GAL1* and the *GAL1* constructs were mapped following 15 and 90 minutes induction with galactose. Mapping was carried out as described previously (30).

Mapping and normalisation of ChIP-seq data in *SET2, RCO1, EAF3* and *SET1* deletion strains. Nucleosome occupancy, H3K14ac and H3K36me3 levels in wild-type and four deletion strains were obtained from recent ChIP-seq data (29). FASTQ files were obtained from ENA and aligned to the yeast genome in the same manner described in (29). Briefly, paired reads were excluded if they defined a fragment less than 130bp or greater than 220. To obtain occupancy levels for a given modification or for nucleosome occupancy itself, the midpoint of a fragment was considered to be the midpoint of a nucleosome. To compare between strains, occupancy at a given bp was determined as the number of nucleosomes overlapping at that bp divided by the total number of nucleosomes in that experiment. H3K14ac and H3K36me3 levels were also normalised to the nucleosome occupancy, in a manner analogous to the H3 normalisation carried out for Fig. 3. Note that this normalisation approach is different to that described in (29). The difference in occupancy at each bp relative to the TSS, between a given deletion strain and the wild-type, was then computed, and an average obtained across all genes for each of the three groups.

1. Mellor, J. and Morillon, A. (2004) ISWI complexes in Saccharomyces cerevisiae. *Biochim Biophys Acta*, **1677**, 100-112.
2. Flavell, S.W., Kim, T.K., Gray, J.M., Harmin, D.A., Hemberg, M., Hong, E.J., Markenscoff-Papadimitriou, E., Bear, D.M. and Greenberg, M.E. (2008) Genome-wide analysis of MEF2 transcriptional program reveals synaptic target genes and neuronal activity-dependent polyadenylation site selection. *Neuron*, **60**, 1022-1038.
3. Burgess, R.J. and Zhang, Z. (2013) Histone chaperones in nucleosome assembly and human disease. *Nat Struct Mol Biol*, **20**, 14-22.
4. Buchler, N.E. and Bai, L. (2011) Chromatin: bind at your own RSC. *Curr Biol*, **21**, R223-225.
5. Bao, W., Behm, D.J., Nerurkar, S.S., Ao, Z., Bentley, R., Mirabile, R.C., Johns, D.G., Woods, T.N., Doe, C.P., Coatney, R.W. *et al.* (2007) Effects of p38 MAPK Inhibitor on angiotensin II-dependent hypertension, organ damage, and superoxide anion production. *Journal of cardiovascular pharmacology*, **49**, 362-368.
6. Bentley, A.R., Raiszadeh, F., Stover, P.J., Hunter, D.J., Hankinson, S.E. and Cassano, P.A. (2010) No association between cSHMT genotypes and the risk of breast cancer in the Nurses' Health Study. *Eur J Clin Nutr*, **64**, 108-110.
7. Massey, A.J., Borgognoni, J., Bentley, C., Foloppe, N., Fiumana, A. and Walmsley, L. (2010) Context-dependent cell cycle checkpoint abrogation by a novel kinase inhibitor. *PLoS One*, **5**, e13123.

8. Chen, X.F., Kuryan, B., Kitada, T., Tran, N., Li, J.Y., Kurdistani, S., Grunstein, M., Li, B. and Carey, M. (2012) The Rpd3 core complex is a chromatin stabilization module. *Curr Biol*, **22**, 56-63.
9. Gilbertson, R.J., Bentley, L., Hernan, R., Junttila, T.T., Frank, A.J., Haapasalo, H., Connelly, M., Wetmore, C., Curran, T., Elenius, K. *et al.* (2002) ERBB receptor signaling promotes ependymoma cell proliferation and represents a potential novel therapeutic target for this disease. *Clinical cancer research : an official journal of the American Association for Cancer Research*, **8**, 3054-3064.
10. Topalidou, I., Papamichos-Chronakis, M., Thireos, G. and Tzamarias, D. (2004) Spt3 and Mot1 cooperate in nucleosome remodeling independently of TBP recruitment. *EMBO J.*, **23**, 1943-1948.
11. Mayer, A., Lidschreiber, M., Siebert, M., Leike, K., Soding, J. and Cramer, P. (2010) Uniform transitions of the general RNA polymerase II transcription complex. *Nat Struct Mol Biol*, **17**, 1272-1278.
12. Luger, K., Mader, A.W., Richmond, R.K., Sargent, D.F. and Richmond, T.J. (1997) Crystal structure of the nucleosome core particle at 2.8A. resolution. *Nature*, **389**, 251-260.
13. Choudhury, A., Nelson, L.D., Teo, M.T., Chilka, S., Bhattarai, S., Johnston, C.F., Elliott, F., Lowery, J., Taylor, C.F., Churchman, M. *et al.* (2010) MRE11 expression is predictive of cause-specific survival following radical radiotherapy for muscle-invasive bladder cancer. *Cancer Res*, **70**, 7017-7026.
14. Kim, H., Erickson, B., Luo, W., Seward, D., Graber, J.H., Pollock, D.D., Megee, P.C. and Bentley, D.L. (2010) Gene-specific RNA polymerase II phosphorylation and the CTD code. *Nat Struct Mol Biol*, **17**, 1279-1286.
15. Basehoar, A.D., Zanton, S.J. and Pugh, B.F. (2004) Identification and distinct regulation of yeast TATA box-containing genes. *Cell*, **116**, 699-709.
16. Huisinga, K.L. and Pugh, B.F. (2004) A genome-wide housekeeping role for TFIID and a highly regulated stress-related role for SAGA in Saccharomyces cerevisiae. *Mol Cell*, **13**, 573-585.
17. Pokholok, D.K., Harbison, C.T., Levine, S., Cole, M., Hannett, N.M., Lee, T.I., Bell, G.W., Walker, K., Rolfe, P.A., Herbolsheimer, E. *et al.* (2005) Genome-wide map of nucleosome acetylation and methylation in yeast. *Cell*, **122**, 517-527.
18. Kirmizis, A., Santos-Rosa, H., Penkett, C.J., Singer, M.A., Vermeulen, M., Mann, M., Bahler, J., Green, R.D. and Kouzarides, T. (2007) Arginine methylation at histone H3R2 controls deposition of H3K4 trimethylation. *Nature*, **449**, 928-932.
19. Venkatesh, S., Smolle, M., Li, H., Gogol, M.M., Saint, M., Kumar, S., Natarajan, K. and Workman, J.L. (2012) Set2 methylation of histone H3 lysine 36 suppresses histone exchange on transcribed genes. *Nature*, **489**, 452-455.
20. Thorvaldsdottir, H., Robinson, J.T. and Mesirov, J.P. (2013) Integrative Genomics Viewer (IGV): high-performance genomics data visualization and exploration. *Briefings in bioinformatics*, **14**, 178-192.
21. Nguyen, T., Fischl, H., Howe, F.S., Woloszczuk, R., Serra Barros, A., Xu, Z., Brown, D., Murray, S.C., Haenni, S., Halstead, J.M. *et al.* (2014) Transcription mediated insulation and interference direct gene cluster expression switches. *eLife*, **3**.
22. Houseley, J., Rubbi, L., Grunstein, M., Tollervey, D. and Vogelauer, M. (2008) A ncRNA modulates histone modification and mRNA induction in the yeast GAL gene cluster. *Mol Cell*, **32**, 685-695.

23. Venters, B.J. and Pugh, B.F. (2009) A canonical promoter organization of the transcription machinery and its regulators in the Saccharomyces genome. *Genome Res*.
24. Kirmizis, A., Santos-Rosa, H., Penkett, C.J., Singer, M.A., Green, R.D. and Kouzarides, T. (2009) Distinct transcriptional outputs associated with mono- and dimethylated histone H3 arginine 2. *Nat Struct Mol Biol*, **16**, 449-451.
25. Guillemette, B., Drogaris, P., Lin, H.H., Armstrong, H., Hiragami-Hamada, K., Imhof, A., Bonneil, E., Thibault, P., Verreault, A. and Festenstein, R.J. (2011) H3 lysine 4 is acetylated at active gene promoters and is regulated by H3 lysine 4 methylation. *PLoS Genet*, **7**, e1001354.
26. Xu, F., Zhang, Q., Zhang, K., Xie, W. and Grunstein, M. (2007) Sir2 deacetylates histone H3 lysine 56 to regulate telomeric heterochromatin structure in yeast. *Mol Cell*, **27**, 890-900.
27. Schulze, J.M., Hentrich, T., Nakanishi, S., Gupta, A., Emberly, E., Shilatifard, A. and Kobor, M.S. (2011) Splitting the task: Ubp8 and Ubp10 deubiquitinate different cellular pools of H2BK123. *Genes Dev*, **25**, 2242-2247.
28. Guillemette, B., Bataille, A.R., Gevry, N., Adam, M., Blanchette, M., Robert, F. and Gaudreau, L. (2005) Variant histone H2A.Z is globally localized to the promoters of inactive yeast genes and regulates nucleosome positioning. *PLoS Biol*, **3**, e384.
29. Chabbert, C.D., Adjalley, S.H., Klaus, B., Fritsch, E.S., Gupta, I., Pelechano, V. and Steinmetz, L.M. (2015) A high-throughput ChIP-Seq for large-scale chromatin studies. *Mol Syst Biol*, **11**, 777.
30. Grange, T. (2008) Sensitive detection of mRNA decay products by use of reverse-ligation-mediated PCR (RL-PCR). *Methods Enzymol*, **448**, 445-466.
